# RNase E biomolecular condensates stimulate PNPase activity

**DOI:** 10.1038/s41598-023-39565-w

**Published:** 2023-08-09

**Authors:** Michael J. Collins, Dylan T. Tomares, Vidhyadhar Nandana, Jared M. Schrader, W. Seth Childers

**Affiliations:** 1 Department of Chemistry, University of Pittsburgh, Pittsburgh, 15260 USA; 2 Department of Biological Sciences, Wayne State University, Detroit, MI 48202 USA

**Keywords:** RNA, RNA metabolism, Bacterial physiology

## Abstract

Bacterial Ribonucleoprotein bodies (BR-bodies) play an essential role in organizing RNA degradation via phase separation in the cytoplasm of bacteria. BR-bodies mediate multi-step mRNA decay through the concerted activity of the endoribonuclease RNase E coupled with the 3′-5′ exoribonuclease Polynucleotide Phosphorylase (PNPase). In vivo*,* studies indicated that the loss of PNPase recruitment into BR-bodies led to a significant build-up of RNA decay intermediates in *Caulobacter crescentus*. However, it remained unclear whether this is due to a lack of colocalized PNPase and RNase E within BR-bodies or whether PNPase’s activity is stimulated within the BR-body. We reconstituted RNase E’s C-terminal domain with PNPase towards a minimal BR-body in vitro to distinguish these possibilities. We found that PNPase’s catalytic activity is accelerated when colocalized within the RNase E biomolecular condensates, partly due to scaffolding and mass action effects. In contrast, disruption of the RNase E-PNPase protein–protein interaction led to a loss of PNPase recruitment into the RNase E condensates and a loss of ribonuclease rate enhancement. We also found that RNase E’s unique biomolecular condensate environment tuned PNPase’s substrate specificity for poly(A) over poly(U). Intriguingly, a critical PNPase reactant, phosphate, reduces RNase E phase separation both in vitro and in vivo. This regulatory feedback ensures that under limited phosphate resources, PNPase activity is enhanced by recruitment into RNase E’s biomolecular condensates.

## Introduction

Biomolecular condensates are liquid-like to gel-like protein assemblies that organize biochemical processes within membraneless compartments in cells^1–3^. Within these protein-rich ensembles, scaffolding proteins mediate weak multivalent protein-protein^4^ and protein-nucleic acid interactions^5^, facilitating phase separation into liquid-like biomolecular condensates. The scaffold also recruits client proteins into these assemblies resulting in functional compartments that include stress granules, p-bodies^6,7^, the nucleolus^6,8^, and signaling complexes^8,9^ within eukaryotic cells.

One of the earliest observations of bacterial biomolecular condensate was polyphosphate granules that are commonly observed as stained granules in cells^10,11^. These polyphosphate granules play vital roles in the fitness of microbes when exposed to nutrient starvation^10,12,13^. At that early stage, it was unclear what mechanisms drive granule formation, as these observations pre-dated the development of the field of biomolecular condensates^2,14^. However, recently, it has been shown that polyphosphate in vitro forms liquid-like assemblies through phase separation mechanisms^15^.

Moreover, it is now known that biomolecular condensates regulate various biochemical processes in bacteria as membraneless organelle-like structures^16,17^. This has opened the door to reconsidering microbial biochemistry in the context of non-membrane-bound compartments. For example, in bacteria, these membraneless compartments have been found to regulate the spatial organization of ABC transporters^18^, single-stranded (ss) DNA-binding proteins^19^, aggresomes^20^, cell division protein FtsZ^21^, BapA amyloid biofilm matrix protein^22^, circadian rhythm associated proteins^23^, and carboxysomes^24,25^. Much like the intersection of nucleic acids and biomolecular condensates in eukaryotes, bacterial biomolecular condensates commonly involve protein scaffolds that sequester nucleic acids as clients^26–30^. One of the best characterized involves the *Caulobacter crescentus (C. crescentus)* RNA degradosome that phase separates into bacterial ribonucleoprotein bodies (BR-bodies) that mediate the rapid decay of RNAs^26,27^.

**Figure 1:**
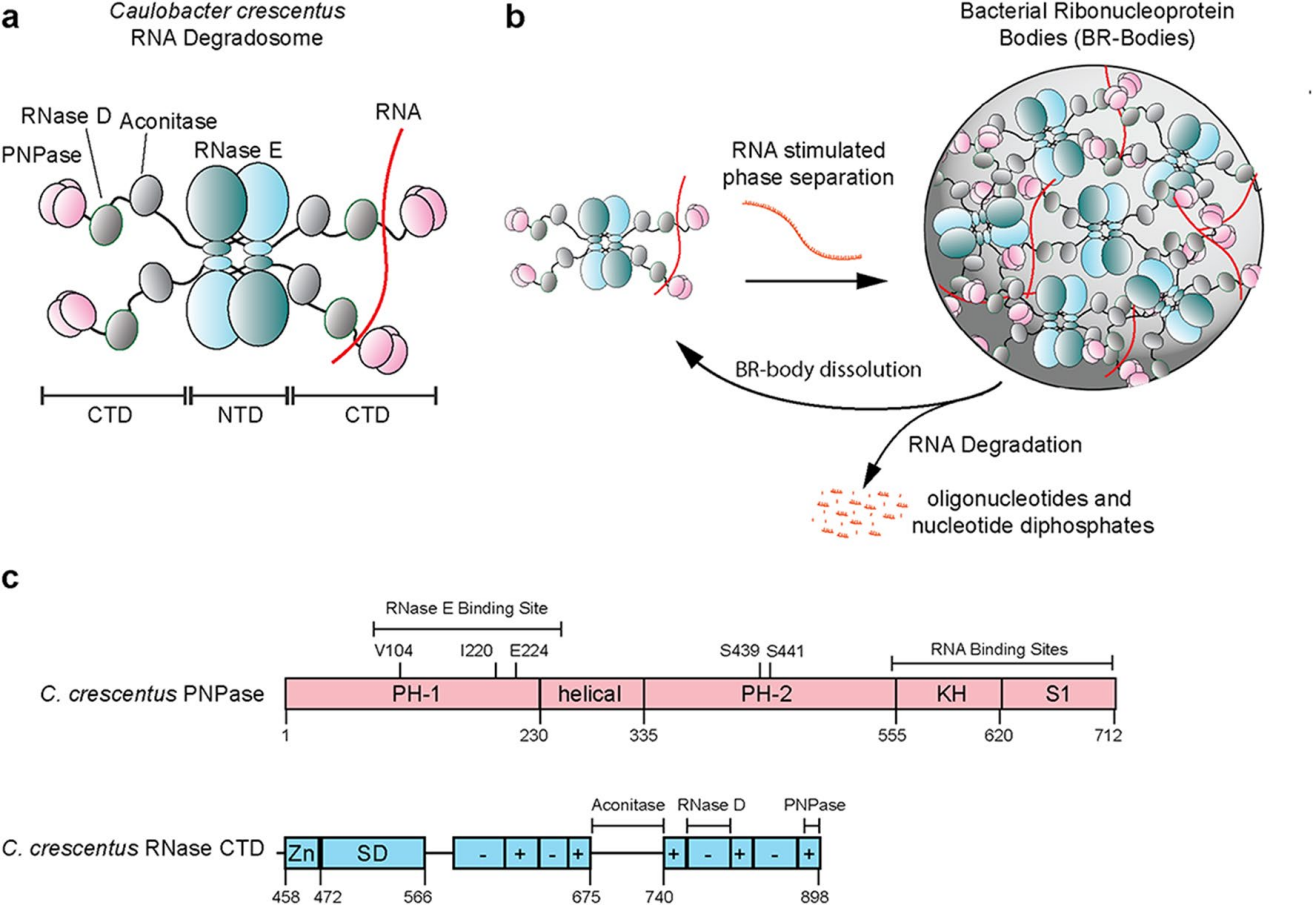
Bacterial Ribonucleoprotein Bodies (BR-bodies) are phase-separated biomolecular condensates of the RNA degradosome. (**a**) The RNA degradosome in *Caulobacter crescentus* consists of RNase E, the primary scaffold that recruits long, unstructured RNAs, PNPase, RNase D, and Aconitase as clients. (**b**) In vivo, phase separation of the degradosome is stimulated by multivalent interactions with the arginine-rich charge blocks^26^ in its C-terminal IDR and long unstructured RNA substrates resulting in the formation of BR-bodies. BR-bodies serve as sites of RNA degradation, in which the endoribonuclease RNase E performs the rate-limiting initial cut of the RNAs. Subsequently, the exoribonuclease PNPase breaks down the RNA intermediates. Breakdown of the RNAs into small oligoribonucleotides and nucleotide diphosphates results in a loss of multivalency and dissolution of the BR-bodies^27^. (**c**) Domain architecture of *C. crescentus* PNPase and RNase E. PNPase is composed of RNase PH domains, the helical domain, the RNA-binding K-homology (KH) domain, and S1 RNA-binding domains^38^. The RNase E C-terminal domain is sufficient for the phase separation of BR-bodies^26^. The RNase E CTD comprises a Zn-link (Zn), a small domain (SD) and an intrinsically disordered region organized as a set of charged blocks.

Across bacterial RNA degradosomes, RNase E is the most common scaffold and frequently binds PNPase, suggesting functional importance to this interaction^31^. In *C. crescentus*, RNase E contains an N-terminal endoribonuclease domain and a C-terminal disordered domain that scaffolds RNAs, PNPase, RNase D, and aconitase (Fig. 1)^32^. Studies have successfully reconstituted the RNA degradosome complex in *E. coli*^33,34^. This includes seminal work by Mackie and colleagues demonstrating that complete degradation of the structured *malEF* substrate required the concerted functions of *E. coli* RNase E, RhlB, and PNPase^35^. Specifically, the PNPase exoribonuclease function is sensitive to the degree of structure within RNA substrates. The RNA helicase RhlB assists by unwinding duplexed RNA, allowing greater accessibility to the co-localized ribonucleases^36^. Polyadenylation of the 3′ end of structured RNA substrates also provides a binding site for PNPase to facilitate the rapid degradation of structured RNAs^37^. However, it is unclear how the phase separation of RNase E regulates the enzymatic function of PNPase activity and its RNA substrate preferences.

Our past studies showed that *C. crescentus* RNase E phase separated and exhibited liquid-like fusion events in vivo and in vitro (Fig. 1)^26^. Protein-rich droplets formed transiently in an RNA-dependent manner, as rifampicin-mediated inhibition of RNA polymerase reduced foci formation of RNase E in vivo^26^. In vivo, BR-body enrichment assays indicate that BR-bodies engage a broad set of RNA substrates with a preference for long and unstructured RNAs^27^. The role of BR-body phase separation in RNA degradation was highlighted by in vivo Rif-seq experiments, where studies indicated that failure to form biomolecular condensates and recruit exoribonuclease clients into BR-bodies, including PNPase, increased global RNA half-lives^27^. Additionally, in an RNase E mutant that phase-separated but could not recruit the RNA degradosome proteins PNPase, Aconitase, or RNase D, a build-up of mRNA decay intermediates was observed suggesting that BR-body associated exoribonucleases coordinate the multi-step mRNA decay pathway^27^. However, these in vivo experiments could not distinguish whether exoribonuclease recruitment to BR-bodies was responsible for the mRNA decay fragment build-up, or whether exoribonucleases have enhanced catalytic activity within BR-bodies. Therefore, here we provide direct in vitro evidence to show PNPase’s localization within RNase E biomolecular condensates stimulates the catalytic rate of PNPase and regulates the RNA substrate specificity of PNPase. We then uncover how levels of a key PNPase enzymatic reactant and nutrient regulate phase separation. This logical wiring of PNPase biochemistry with RNase E phase separation provides a way to regulate RNA decay processes in fluctuating phosphate nutrients.

## Results

### Recruitment of PNPase into the RNase E droplets requires the C-terminal binding site

To investigate the recruitment of PNPase into RNase E droplets, we purified a PNPase active site mutant (PNPase-S439A/S440A/S441A-mCherry) that lacks ribonuclease activity called PNP-ASM-mCherry. Use of the PNPase active site mutant minimizes the potential impact of PNPase’s breakdown of RNA upon co-localization within the RNase E droplets. In addition, we purified the RNase E C-terminal domain (residues 451–898), called Rnase E CTD, which is sufficient for phase separation^26^ and contains the C-terminal PNPase binding site (residues 889–898). These protein constructs allow us to consider how RNase E’s phase separation properties impact PNPase activity.

We visualized each mixture via phase contrast and fluorescence microscopy imaging to determine if the purified RNase E CTD-YFP could recruit PNPase-mcherry as a client. We then calculated the fluorescence intensity ratio in the concentrated versus the dilute phase for each assembly, which we term partitioning ratio (PR). Differences in the degree of protein enrichment in the dense phase directly vary the fluorescence intensity. However, the unique chemical environment or these assemblies may alter the refractive index or impact the quantum yield of fluorescent proteins. In addition, the crowding of fluorescent proteins nearby may lead to quenching the fluorescence signal. Therefore, the PR reflects the combinations of these effects.

**Figure 2:**
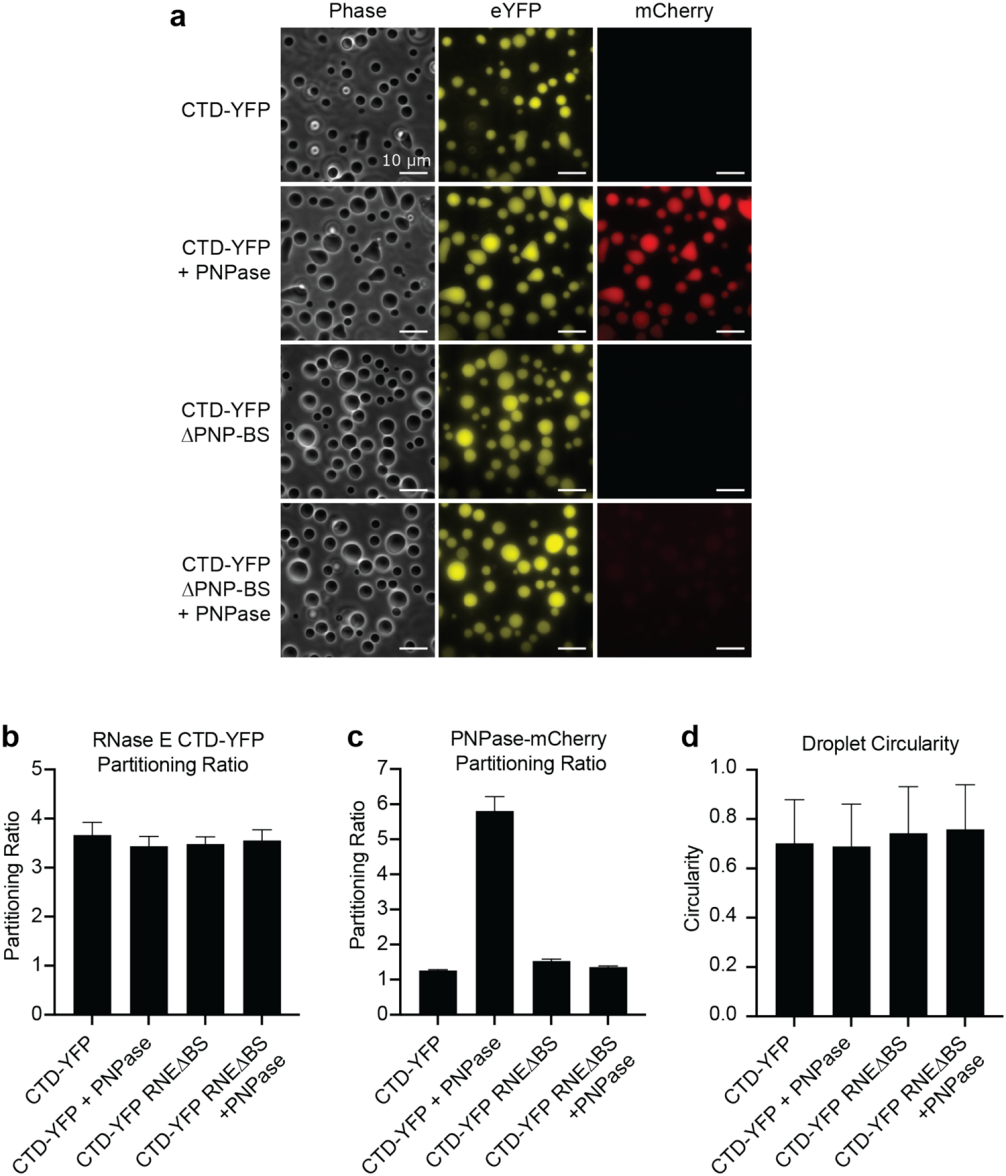
PNPase partitions into RNase E biomolecular condensates by interacting with a C-terminal binding site on RNase E-CTD. We found that PNPase enrichment into RNase E condensates was mediated by a specific protein–protein interaction. (**a**) Phase-contrast and fluorescence microscopy images of RNase E biomolecular condensates. RNase E and RNase E-∆PNP-BS contained a C-terminal eYFP tag and were present at 20 µM, and PNPase-ASM (active site mutant) contained a C-terminal mCherry tag and was at a concentration of 1 µM. Scale bar is 10 µm. (**b**) Average partitioning ratios and standard deviations are presented for RNase E. There is no statistically significant difference between any PRs (*p* > 0.05). (**c**) Average partitioning ratios and standard deviations are presented for PNPase. Amongst experiments, PNPase is only significantly recruited into RNase E biomolecular condensates (*p* < 0.001). In all other cases, PNPase does not significantly partition into RNase E biomolecular condensates (*p* > 0.05). (**d**) Average droplet circularity and standard deviations. See the methods section for the droplet circularity calculation formula. Data represent the average and standard deviation of n > 300 droplets from three replicates of images.

We found 20 µM RNase E CTD-YFP in a buffer containing 10 mM MgCl_2_ and 100 mM NaCl phase-separated into protein-rich biomolecular condensates with a 3.6 ± 0.3 partitioning ratio (Fig. 2a,b). The addition of the 5 µM PNPase-ASM client had no impact on the RNase E CTD-eYFP partitioning ratio (*p* = 0.13) (Fig. 2b). In comparison, the PNPase-ASM-mCherry client did not separate into a protein-rich phase under the same conditions. However, the co-assembly of PNPase with RNase E led to the recruitment of PNPase into the RNase E droplets with a partitioning ratio of 5.7 ± 0.7 (Fig. 2c).

We next considered if specific protein–protein interactions mediated the recruitment of PNPase into the RNase E condensates. We, therefore, tested if an RNase E variant, RNase E-∆PNP-BS, lacking the 10 C-terminal residues that bind PNPase^38^, could recruit PNPase. Individually, both RNase E-CTD and RNase E CTD∆PNP-BS phase-separated into biomolecular condensates (Fig. 2a) with a partitioning ratio of 3.6 ± 0.3 and 3.5 ± 0.4, respectively (Fig. 2b). This indicates that the C-terminal PNPase binding site residues are unnecessary for RNase E’s homotypic phase separation. However, the RNase E CTD∆PNP-BS variant displayed reduced recruitment of PNPase with a partitioning ratio of 1.4 ± 0.1, which was significantly less than RNase E CTD recruitment of PNPase (*p* < 0.001) (Fig. 2c). Thus, PNPase requires the 10 C-terminal residues of RNase E for enrichment within RNase E condensates.

To understand the contributions of weak fluorescent protein interactions on client recruitment to the RNase E biomolecular condensates and droplet morphology, free mCherry was incubated with RNase E-CTD or RNase E CTD∆PNP-BS. The partitioning ratio of mCherry was 1.2 ± 0.1 in RNase E droplets and 1.2 ± 0.1 in RNase E CTD∆PNP-BS droplets (Fig. S1a–c). This indicates weak enrichment with PNPase-mCherry partitioning ratios in the range of 1.0–1.3 may be due to weak fluorescent protein interactions. In contrast, enhanced recruitment beyond that amount requires a specific binding site. However, we observed that adding free mCherry leads to changes in RNase E CTD-YFP circularity (Fig. S1d). In comparison, the addition of unlabeled PNPase did not lead to observable changes in circularity (Fig. S1e,f). This suggests weak fluorescent protein interactions can contribute to RNase E condensate morphology changes.

### PNPase triple mutant disrupts recruitment into RNase E biomolecular condensates

**Figure 3:**
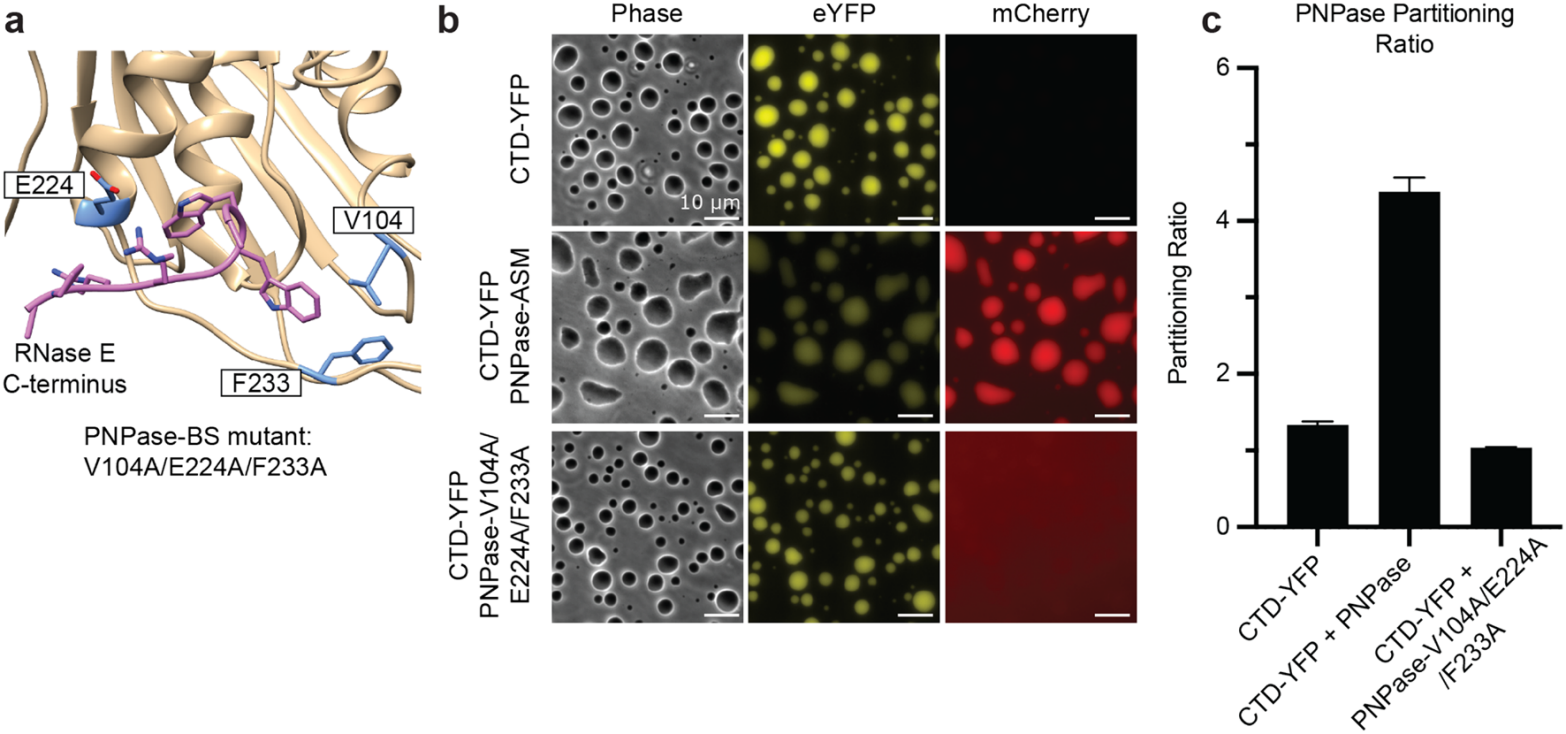
Identification of PNPase residues critical for interaction with RNase E. Here, we found that a PNPase variant containing mutations at the RNase E-PNPase interface was not recruited as a client into the RNase E biomolecular condensates. This indicates that a specific RNase E-PNPase interaction mediates PNPase enrichment into the RNase E biomolecular condensates. (**a**) The co-crystal structure of *C. crescentus* RNase E C-terminal peptide (PDB 4AIM)^38^ bound to PNPase highlights the critical protein–protein interaction site. (**b**) Phase-contrast and fluorescence microscopy images of RNase E biomolecular condensates mixed with PNPase-ASM-mCherry or the PNPase-V104A/E224A/F233A-mCherry variant. The scale bar is 10 µm. (**c**) Average partitioning ratios and standard deviations are presented for PNPase and the PNPase-V104A/E224A/F233A-mCherry variant. Amongst experiments, PNPase is only significantly recruited into RNase E biomolecular condensates (*p* < 0.001). The PNPase-V104A/E224A/F233A-mCherry variant does not significantly partition into RNase E biomolecular condensates (*p* > 0.05). Data represent the average and standard deviation of three replicates.

Deleting the C-terminal residues of RNase E may also alter the multivalent contacts that mediate RNase E phase separation. Therefore, we considered if mutations within PNPase could disrupt recruitment into the wild-type RNase E CTD condensates. RNase E binds to a hydrophobic pocket on the external surface of the catalytic core of PNPase facilitated by residues G896, W897, and W898^38^. These RNase E residues interact directly with PNPase’s V104, I220, E224, and F233 residues (Fig. 3a). We hypothesized that mutating these PNPase residues would diminish PNPase recruitment into the RNase E biomolecular condensates. Therefore, we cloned and purified a PNPase variant, PNPase-V104A/E224A/F233A, to disrupt interactions between RNase E and PNPase. When incubated with RNase E, incorporation of PNPase-V104A/E224A/F233A into the RNase E condensates was diminished from a PR of 4.4 ± 0.2 (PNPase-ASM) to 1.0 ± 0.1 (PNPase-V104A/E224A/F233A) (Fig. 3b,c). Thus, PNPase utilizes a specific binding site (Fig. 3a) for enrichment into RNase E biomolecular condensates.

### A binding site mimetic peptide disrupts PNPase recruitment into RNase condensates

We next considered if the short peptide representing the 10 C-terminal residues of RNase E, EKPRRGWWRR, could disrupt PNPase association with RNase E^32^. From here on, we refer to this as the GWW peptide. We observed that when PNPase was incubated with 100 µM GWW peptide before adding RNase E to stimulate droplet formation, the PNPase PR decreased from 2.8 ± 0.2 to 1.7 ± 0.2. We found that the GWW peptide did not disrupt RNase E phase separation (Fig. S2a,b), but it caused a 5% reduction (p < 0.001) in RNase E droplet circularity (Fig. S2e). Interestingly, GWW peptide concentrations of 100 µM or above resulted in a patchy appearance of PNPase-mCherry within the RNase E protein-rich droplets (Figs. S2d, S3a). These results indicate that the GWW peptide outcompetes the C-terminus of RNase E for binding with PNPase, lowering the amount of PNPase associated with the RNase E condensates.

We next considered if the GWW peptide’s disrupting functions require a specific protein sequence as indicated in the RNase E-PNPase co-crystal structure (Fig. 3a) or just requires a peptide of specific amino acid composition. We thus tested the ability of a scrambled version of the GWW peptide to diminish PNPase’s partitioning into RNase E droplets. The GWW scrambled peptide retains the same amino acid composition but a different sequence, namely KRWREWGRPR. This scrambled peptide separates the pair of adjacent tryptophan residues, which interacts with PNPase. We found that titration of GWW peptide from 0 to 200 µM into an RNase E-PNPase droplet mixture disrupts PNPase recruitment in a dose-dependent manner. In comparison, the GWW scrambled peptide does not disrupt PNPase recruitment (Fig. S3b,c). These results suggest the potential of short peptides functioning as inhibitors to disrupt PNPase client recruitment into RNase E condensates.

### RNA is not sufficient for PNPase recruitment into RNase E biomolecular condensates

We next considered if RNA clients of RNase E can recruit additional RNA binding proteins that do not directly associate with RNase E. Such recruitment of non-clients would impact a BR-body's capability to control its composition to facilitate mRNA decay rather than other RNA modification biochemistry. Given that RNase E and PNPase can both bind RNA^38^, we interrogated whether poly(A), a preferred substrate for PNPase, could promote PNPase association with RNase E condensates lacking the PNPase binding site. We found that poly(A) was insufficient to drive PNPase accumulation in RNase E biomolecular condensates lacking the C-terminal binding site at poly(A) concentrations ranging from 25 to 100 ng/µL (Fig. S4a–c). These results suggest that poly(A) has a poor capacity to recruit PNPase and that the protein–protein interaction with the C-terminus of RNase E is likely the main driver of PNPase recruitment into RNase E condensates.

### RNase E condensates enhance PNPase nuclease activity against poly(A)

**Figure 4:**
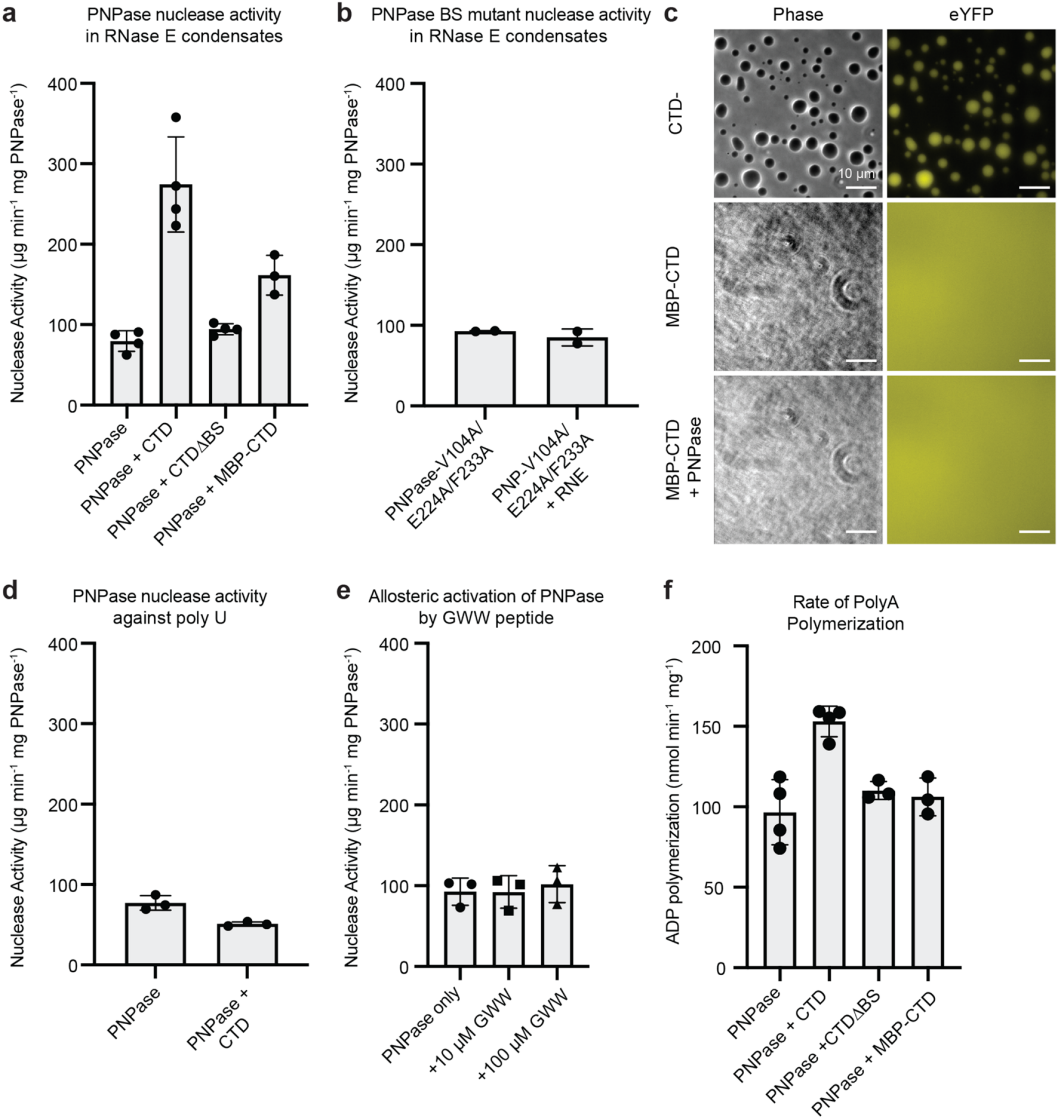
RNase E biomolecular condensates stimulate the ribonuclease functions of PNPase. Here we found that PNPase recruitment into the RNase E condensates stimulated PNPase’s degradation of poly(A) substrates. In addition, we found the addition of the maltose-binding protein (MBP) tag to RNase E solubilized the RNase E condensates. We found that MBP-RNase E did not stimulate PNPase ribonuclease functions to the same level, suggesting a role of RNase phase-separated environment upon PNPase stimulation. (**a**) Rate of ribonuclease activity towards 25 ng/µL poly(A) for 5 µM PNPase alone, 5 µM PNPase and 20 µM RNase E, 5 µM PNPase and 20 µM RNase E-∆BS (RNase E lacking the 14 C-terminal amino acids to which PNPase binds), and 5 µM PNPase and 20 µM MBP-RNase E (Maltose Binding Protein-RNase E fusion which cannot phase separate). (**b**) Ribonuclease activity of PNPase-V104A/E224A/F233A-mCherry (PNPase triple mutant lacking the ability to bind RNase E) and PNPase-V104A/E224A/F233A-mCherry mixed with RNase E. No significant rate increase was observed when RNase E was added (*p* > 0.05). (**c**) Phase contrast and fluorescence microscopy images of RNase E CTD-YFP versus RNase E MBP-CTD-YFP. Fusion of maltose-binding protein (MBP) significantly reduced the phase separation properties of RNase E. (**d**) Ribonuclease activity towards 25 ng/µL poly(U) for five µM PNPase alone, or 5 µM PNPase and 20 µM RNase E. No significant difference in rate was observed (*p* > 0.05). (**e**) Ribonuclease activity of 5 µM PNPase after incubation with 10 µM or 100 µM GWW peptide. No significant difference in rate was observed (*p* > 0.05). (**f**) RNase E droplet formation stimulates PNPase polymerase activity. The rate of PNPase polymerization of ADP into poly(A) is faster in the presence of RNase E droplets than with PNPase alone. However, PNPase activity is not enhanced when the recruitment of PNPase to RNase E droplets is diminished (RNase E CTD∆BS) or when RNase E cannot form droplets (RNase E MBP-CTD). Data represent the average and standard deviation of at least three trials.

Past biochemical studies of PNPase have shown that PNPase selectively degrades unfolded substrates, whereas folded RNA hairpins can inhibit PNPase function. Folded RNA substrates require the helicase activity of RhlB or polyadenylation to become suitable substrates for PNPase^35^. We utilized unstructured poly(A) and poly(U) RNA to minimize RNA substrate folding effects that limit PNPase enzymatic functions. We mixed 20 mM Rnase E CTD-YFP, 5 µM PNPase-mCherry, and 25 ng/µL of poly(A) to assay PNPase's exoribonuclease activity. After initiating reactions with poly(A), we tracked poly(A) RNA degradation using denaturing PAGE gels stained with SYBR Gold RNA stain. In the absence of RNase E, PNPase degraded poly(A) at a rate of 80 ± 13 µg min^−1^ (mg PNPase)^−1^ (Fig. 4a). This rate of degradation by *C. crescentus* PNPase is about tenfold lower than past reports of *E. coli* PNPase enzymatic functions^39^. These differences may be due to intrinsic activity differences between the two PNPase homologs or differences in the temperature during the assays (room temperature versus 37 °C).

We next assessed how the phase-separated environment of RNase E impacted PNPase function. The PNPase-RNase E co-assembly degraded poly(A) at a rate of 274 ± 60 µg min^−1^ (mg PNPase)^−1^. This corresponds to a 3.4-fold enhancement of the rate of poly(A) degradation over PNPase alone (*p* < 0.001) (Fig. 4a).

It's possible that RNase E condensate may have a non-specific effect on PNPase (e.g., by increasing the bulk viscosity of the solution). To consider this possibility, we tested how the lack of PNPase recruitment into the RNase E CTD-YFP condensates would impact PNPase function. Upon mixing PNPase with an RNase E variant lacking the PNPase binding site, RNase E-∆PNP-BS, we observed a poly(A) degradation rate of 94 ± 7 µg min^−1^ (mg PNPase)^−1^. The observed 1.2-fold enhancement which was not significantly different from PNPase alone (*p* = 0.92) (Fig. 4a). This result indicates that a direct interaction between RNase E and PNPase is required for the 3.4-fold rate enhancement of poly-A degradation.

As opposed to disruption of direct recruitment PNPase in the RNase E-∆PNP-BS variant, removing these PNPase binding residues may alter the multivalent contacts of RNase E that mediate phase separation. This structural re-organization of RNase E might indirectly impact the condensate ability to stimulate PNPase function. We, therefore, considered the activity of the PNPase-V104A/E224A/F233A variant that does not co-localize with RNase E CTD (Fig. 3). Upon mixing PNPase-V104A/E224A/F233A with RNase E CTD condensates, we observed a poly(A) degradation rate of 85 ± 11 µg min^−1^ (mg PNPase)^−1^, which was not significantly different from the rate of PNPase-V104A/E224A/F233A alone of 93 ± 1 µg min^−1^ (mg PNPase)^−1^ (*p* = 0.40) (Fig. 4b). Therefore, RNase E does not sequester PNPase-V104A/E224A/F233A as a client (Fig. 3c) and subsequently does not enhance the catalytic functions of PNPase-V104A/E224A/F233A (Fig. 4b).

In summary, we have considered the impact of the disruption of the RNase E-PNPase interaction through both RNase E variants (Fig. 4a) and PNPase variants (Fig. 4b). Both variants lead to a loss of PNPase recruitment into the RNase E condensates and a failure to stimulate PNPase’s breakdown of poly (A). These results suggest that the specific protein–protein interaction of RNase E’s C-terminal residues enhances, directly or indirectly, the ribonuclease activity of PNPase.

One consideration in these assays was the ratio of RNase E:PNPase. In *C. crescentus,* the copy numbers of RNase E range from 10 to 20 µM, while PNPase ranges from 35 to 45 µM. We examined assays at a 20:5 ratio of RNase E: PNPase to bias PNPase to be in its condensate-bound form. Additionally, lower PNPase concentrations ensure that the initial reaction rates are sufficiently slow for enzymatic characterization to distinguish condensate bound and free forms of PNPase. We anticipate varied stoichiometry of this complex will also lead to optimizing this rate enhancement to ensure each RNase E scaffold engages one RNA substrate and one PNPase client.

### RNase E condensates selectively enhance the degradation of poly(A) but not poly(U)

One key model of how condensates tune the activity of enzymes is by altering substrate specificity through co-localization and exclusion effects. The two RNA binding sites within the CTD, AR1 and AR2, present additional RNA binding sites that may influence the substrate preference of the client enzyme PNPase. We investigated if the phase-separated environment of RNase E regulates the substrate specificity of PNPase by comparing two unstructured substrates: poly(A) versus poly(U). Free PNPase degrades poly(U) at a rate of 77 ± 9 µg min^−1^ (mg PNPase)^1^, which is not significantly different from the rate at which PNPase degrades poly(A) (*p* = 0.99) (Fig. 4d). This indicates that *C. crescentus* PNPase alone has no substrate preference for poly(A) or poly(U).

In comparison, we observed that the addition of RNase E to PNPase led to a decreased poly(U) degradation rate of 51 ± 3 µg min^−1^ mg PNPase^−1^ (Fig. 4d). Therefore, unlike the 3.6-fold enhancement of poly(A) degradation mediated by RNase E, we observed a 34% reduction in poly(U) degradation upon RNase E addition. Remarkably, PNPase degrades poly(A) 5.4-fold faster than poly(U) in the presence of RNase E condensates. These results indicate that in vitro*,* the chemical environment of RNase E alters PNPase’s substate selectivity. Interestingly, the enhanced selectivity of poly(A) over poly(U) correlates with the role of 3′ polyadenylated tails in accelerating RNA decay in vivo^37^.

We next considered if the enhanced selectivity of poly(A) over poly(U) was rooted in a difference in the enrichment of poly(A) versus poly(U) within the RNase E condensates. We observed that Cy5-poly(A) and Cy5-poly(U) displayed similar enrichment of about 1.2-fold into the RNase E biomolecular condensates versus the dilute environment (Fig. S5a–c). Due to PNPase’s RNA binding capabilities, we considered how the addition of PNPase to the RNase E-RNA mixture would impact poly(A) and poly(U) recruitment. We observed that adding PNPase did not significantly increase Cy5-Poly(U) recruitment. In contrast, Cy5-poly(A) displayed an increased PR of 1.6 when co-assembled with the RNase E-PNPase biomolecular condensates (Fig. S5a–c). This increased enrichment suggests that the substrate selectivity of PNPase in the presence of RNase E is impacted by the increased poly(A) enrichment relative to poly(U).

Notably, we also observed some changes in droplet morphology in the presence of both RNA substrates and PNPase. The circularity of RNase E condensates with poly(A) or poly(U) was not significantly different than RNase E condensates alone (p = 0.87, p = 0.94, respectively) (Fig. S5d). In comparison, the addition of PNPase significantly decreased the average droplet circularity of RNase E condensates enriched with poly(A) by 15% (p < 0.001) and enriched with poly(U) by 17% (p < 0.001) (Fig. S5d). This suggests that there may also be contributions in how poly(A) and poly(U) impact the viscosity of the RNase E-PNPase condensates.

### RNase E biomolecular condensates enhance PNPase polymerization of poly(A)

In addition to its primary function as a ribonuclease against single-stranded RNA, PNPase also catalyzes the polymerization of nucleotide diphosphates to form single-stranded RNA^39,40^. We, therefore, considered how RNase E’s phase-separated environment impacts PNPase’s polymerase activity. PNPase alone polymerized 100 ± 20 mmol ADP min^−1^ mg PNPase^−1^ while PNPase in the presence of RNase E condensates polymerized ADP at a rate of 153 ± 9 mmol min^−1^ mg PNPase^−1^ (p < 0.001) (Fig. 4f). As a control, we considered if PNPase’s polymerase activity could be stimulated by RNase E CTD variants that do not colocalize with the RNase E condensates, RNase E CTD∆PNP BS. We found that PNPase polymerized ADP at a rate of 110 ± 6 mmol min^−1^ mg PNPase^−1^ in the presence of condensates formed from RNase E-∆PNP BS, which was not significantly different from the polymerase activity of PNPase alone (p = 0.66). Similarly, in the presence of MBP-RNase E, which is unable to form condensates, PNPase polymerized ADP at a rate of 106 ± 12 mmol ADP min^−1^ mg PNPase^−1^ and is not significantly different from PNPase alone (p = 0.87). Therefore, the phase-separated environment has a mild 1.5-fold impact on PNPase polymerase activity.

The function of PNPase as ribonuclease or polymerase will depend upon the accessibility of phosphate, RNA substrates, and ADP substrates. Interestingly, the presence of RNase E droplets stimulates PNPase nuclease activity by 3.4-fold but only stimulates polymerase activity by 1.5-fold. This indicates that the condensate biochemical environment has a greater impact on ribonuclease functions than polymerase functions. This may be due to poor enrichment of the ADP substrate in comparison to poly(A) substrate (Figs. 4f, S5). Moreover, in vivo, we have observed that the presence of RNA stimulates BR-body formation^26^. These data suggest that the observed RNase E biomolecular condensates in cells are sites of RNA degradation instead of sites of polymerase activity.

### RNase E’s scaffolding and phase separation plays a key role in PNPase regulation

We next considered three mechanisms for how RNase E could stimulate PNPase phosphorylase activity. The first is that RNase E’s C-terminal binding site allosterically activates PNPase. In the second model, RNase E brings PNPase and poly(A) nearby via scaffolding, stimulating enhanced exoribonuclease activity. Finally, a third model considers the unique chemical environment of biomolecular condensates that concentrates both poly(A) and PNPase. This third model builds upon the scaffolding effect to include the impact of a higher concentration of PNPase and poly(A) in the RNase E biomolecular condensates, thereby increasing the kinetics of PNPase through mass action.

To test allostery without phase separation and scaffolding, we incubated PNPase with the GWW peptide from RNase E for 30 min before measuring exonuclease activity against poly(A). There was no significant difference in PNPase activity when 10 or 100 µM of the peptide was added (*p* > 0.05) (Fig. 4e), indicating that the RNase E GWW peptide does not allosterically activate PNPase. While this known direct interaction between the GWW peptide and PNPase does not stimulate PNPase function, the full-length RNase E may make additional unknown contacts with PNPase that mediate allosteric regulation.

To examine the impact of scaffolding with diminished phase separation, we used a maltose-binding protein fusion of RNase E’s CTD, called RNase E MBP-CTD. Rnase E MBP-CTD retains the RNA binding and PNPase binding sites but does not phase separate in vitro (Fig. 4c). This RNase E MBP-CTD retains the proximal binding sites to place the poly (A) and PNPase in close proximity without the increased concentration of poly (A) and PNPase afforded by phase separation. The PNPase activity in the presence of MBP-RNase E was elevated twofold over PNPase alone to 160 ± 20 µg min^−1^ (mg PNPase)^−1^ (*p* < 0.05) (Fig. 4a). Analysis of these constructs suggests that scaffolding in the dilute phase increases PNPase activity twofold.

In comparison, phase separation of the RNase E-PNPase scaffolded complex yields a 3.4-fold enhancement and an additional 1.7-fold increase in PNPase activity over scaffolded PNPase (Fig. 4a). Therefore, scaffolding alone in the dilute phase can also partially enhance PNPase activity, as highlighted in earlier studies of RNase E’s scaffolding functions^34,35^. Notably, one caveat for using a MBP fusion is that the bulky MBP tag may exert other steric effects upon the RNase E-PNPase complex in addition to diminished phase separation.

### Magnesium and phosphate impact RNase E droplet formation

**Figure 5:**
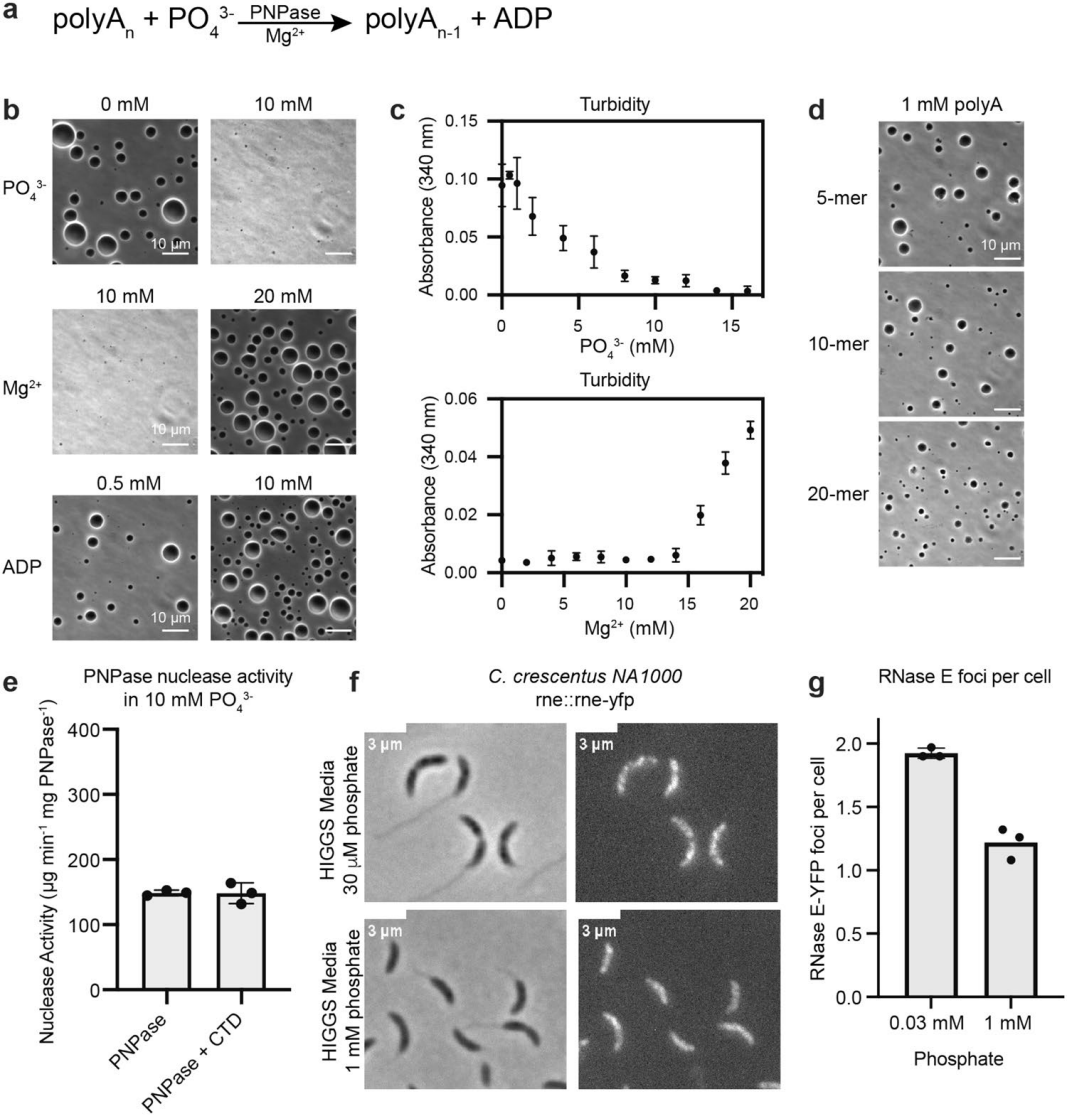
High sodium phosphate or low magnesium chloride levels dissolve the RNase E biomolecular condensates. *Caulobacter crescentus* lives in diverse environmental conditions with fluctuating nutrients such as magnesium and phosphate. We found that low phosphate levels stimulate RNase E phase separation in vivo and in vitro, whereas high levels of phosphate lead to the dissolution of the RNase E biomolecular condensates. (**a**) We considered the logic of how PNPase’s biochemistry is connected to RNase E’s phase separation. PNPase utilizes a magnesium ion cofactor to catalyze the phosphorolysis of a single nucleotide (AMP) by adding inorganic phosphate to release nucleoside diphosphates (ADP). (**b**) Phase-contrast images of RNase E biomolecular condensates in 0 mM or 10 mM sodium phosphate, 10 mM or 20 mM magnesium chloride, and 0.5 mM or 10 mM ADP. Scale bar is 10 µm. (**c**) Turbidity measurement at 340 nm of 20 µM RNase E in a titration of magnesium chloride or sodium phosphate. (**d**) Phase-contrast images of RNase E biomolecular condensates mixed with 1 mM poly(A) 5-mer, 10-mer, or 20-mer. (**e**) Rate of ribonuclease activity towards 25 ng/µL poly(A) for 5 µM PNPase alone or 5 µM PNPase and 20 µM RNase E in 10 mM sodium phosphate that does not form phase-separated assemblies. Scale bar is 10 µm. (**f**) Effect of increased phosphate on BR-bodies in vivo. (**g**) The average number of foci when cells are grown in Higgs medium supplemented with 30 µM and 1 mM phosphate. Error bars represent the standard deviation from three biological replicates.

So far, we have considered how the phase-separated environment of RNase E regulates PNPase enzymatic functions (Fig. 4). However, it is also critical to consider how the biochemistry associated with PNPase impacts the phase separation of RNase E. In a magnesium-dependent manner, PNPase cleaves single-stranded RNA substrates using phosphate to attack the phosphodiester linkage at the 3′ terminus resulting in nucleoside diphosphate products (Fig. 5a). Do magnesium and phosphate ions influence RNase E’s phase properties? This may also be critical, as *Caulobacter crescentus* grows in diverse natural environments that vary in phosphate nutrient availability. Above a critical concentration of 12 mM MgCl_2_, we observed that 20 µM Rnase E CTD-YFP phase-separated into biomolecular condensates (Figs. 5b,c, S6a). In contrast, in the presence of 20 mM MgCl_2_, the addition of phosphate above 10 mM dissolved the RNase E biomolecular condensates (Figs. 5b,c, S6a). Thus, the RNase E-PNPase complex at 20 µM RNase E CTD-YFP forms a protein-rich phase in 20 mM MgCl_2_ with sodium phosphate levels that range from 0 to 8 mM (Fig. S6a). This sensitivity to high sodium phosphate is consistent with our past observations that sodium chloride concentrations that exceed 250 mM dissolve RNase E biomolecular condensates^26^.

We proposed that protein–protein interactions between RNase E’s positively and negatively charged blocks play a key role in RNase E’s homotypic phase separation. Whereas RNase E’s positively charged block interaction with negatively charged RNA regulates its RNA-stimulated phase separation. Therefore, we speculate that the negatively charged phosphates interact with the positively charged blocks in a way that competes with and disrupts key multivalent interactions that drive RNase E’s phase separation. Phosphate ions may also directly compete for binding to the same interaction motifs, or phosphate ions may alter RNase E’s conformation to prevent multivalent interactions. Delineation of these models will require future structural studies to identify the multivalent interaction that mediates RNase E phase separation.

The addition of sodium phosphate increases the ionic strength of the solution, which can strongly influence electrostatic interactions between RNase E molecules and alter the ability to form droplets. To compare the effect of sodium phosphate in a solution of constant ionic strength, we titrated NaCl, NaH_2_PO_4_/Na_2_HPO_4_, and Na_2_SO_4_ to match the ionic strength range of sodium phosphate (Fig. S7). While 6 mM sodium phosphate was sufficient to drive the dissolution of RNase E droplets, the equivalent ionic strength of sodium chloride was not sufficient to dissolve RNase E droplets. Interestingly, this effect is not generally applicable to all divalent anions since sodium sulfate's equivalent ionic strength was insufficient to dissolve RNase E droplets. To further decouple the effect of phosphate from sodium, we titrated the potassium salts of chloride, phosphate, and sulfate. Again, potassium phosphate drove the dissolution of RNase E droplets, while the equivalent ionic strength of potassium chloride or potassium sulfate did not.

### RNase E fails to stimulate PNPase functions in high phosphate buffers

Given the impact of high phosphate on the dissolution of RNase E biomolecular condensates, we also hypothesized that RNase E would no longer stimulate PNPase’s activity under high phosphate conditions. Indeed, in 10 mM sodium phosphate, we found that the activity of PNPase was unaltered by adding RNase E (Fig. 5e). This adds an additional line of evidence that RNase E’s phase separation properties are critical to its ability to enhance PNPase functions. Overall, the sensitivity of RNase E’s phase separation and PNPase regulation capabilities suggest that cytosolic phosphate concentrations may impact BR-body formation in vivo.

In *E. coli*, orthophosphate concentrations range from 1 to 30 mM depending on the availability of a carbon source^41^. In comparison, within *Caulobacter crescentus,* phosphate-poor conditions range from 0 to 50 µM, while phosphate-rich conditions are approximately 1 mM. Therefore, we examined if variations in phosphate nutrients impacted BR-body formation in *C. crescentus* with RNase E-YFP expressed as the sole copy by its native promoter^26^. We found that RNase E-YFP formed 1.9 ± 0.03 BR-bodies/cell under phosphate nutrient-poor conditions. In contrast, we observed a decrease to 1.2 ± 0.10 BR-bodies/cell in phosphate-rich conditions (Fig. 5f,g). This is consistent with our in vitro observations that higher phosphate levels dissolve the RNase E protein-rich droplets. Indeed, our in vitro titration experiments (Fig. 5c) suggest that even higher phosphate levels may further diminish BR-body formation.

### PNPase RNA degradation products do not dissolve RNase E biomolecular condensates

Given that PNPase’s ribonuclease activity results in the production of NDPs, we examined if ADP could dissolve the RNase E biomolecular condensates in vitro. We found that the addition of ADP from 0.5 to 10 mM had no impact on the phase separation properties of RNase E CTD-YFP (Figs. 5b, S6b,d). This indicates that ADP products of PNPase nuclease activity do not directly regulate RNase E’s phase separation. The ability to avoid biomolecular condensate dissolution at high levels of ADP may be critical to maintaining the enhanced RNA decay within these assemblies.

It is also possible that PNPase stalled on a reaction substrate could release a short oligonucleotide product. We considered the possibility that short oligoribonucleotides might be more effective at dissolving RNase E droplets than ADP. Poly(A) oligos of lengths 5, 10, and 20 nucleotides were incubated at 500 µM and 1000 µM with 20 µM RNase E to examine how short RNA products affect RNase E’s phase separation properties (Figs. 5d, S6c,e). The addition of these short poly(A) oligos did not cause the dissolution of RNase E droplets. These results indicate that the ribonuclease activity products of PNPase do not cause RNase E biomolecular condensates to dissolve.

## Discussion

**Figure 6:**
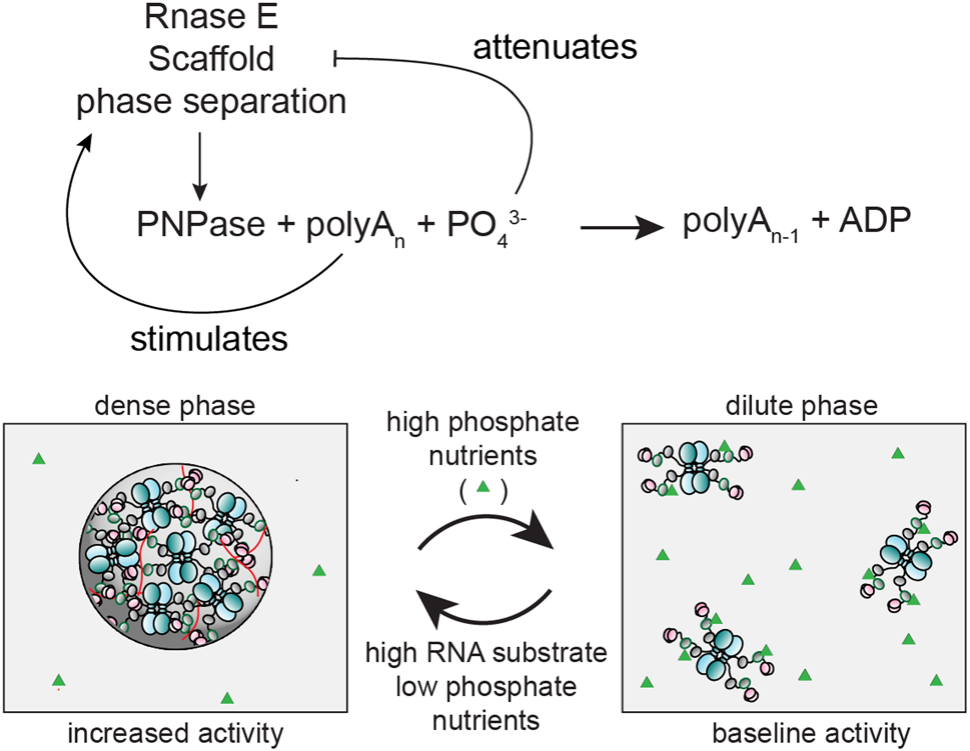
The logical wiring of RNase E condensate phase separation with PNPase enzymatic functions. Our studies found that RNase E phase separation is sensitive to a critical reactant, phosphate. High phosphate nutrients dissolve RNase E CTD condensates in vitro, diminishing BR-body formation in vivo. Low phosphate nutrients stimulate phase separation and PNPase activity when the concentration of this critical phosphate nutrient is low. Thus the availability of phosphate nutrients provides a negative feedback loop upon the regulation of RNase E condensate formation and function. Our previous work has shown that RNA substrate availability stimulates BR-body formation. Therefore, RNA substrates provide a positive feedback loop that provides on-demand highly active RNase E condensates.

This study investigated how RNase E regulates PNPase activity and how the biochemistry associated with the PNPase client impacts RNase E phase separation. We found that critical cofactors and substrates of PNPase, such as divalent magnesium and phosphate ions, regulated the phase separation properties of RNase E protein-rich biomolecular condensates in vitro (Fig. 6). Interestingly, model system studies of arginine-rich peptides mixed with RNA showed that various divalent ions could alter the material properties and the switch from heterotypic to homotypic phase separation^42^. Our results suggest that the availability of magnesium and phosphate nutrients in the environment may alter the material properties and composition of BR-bodies in *C. crescentus*.

Our current simplified system of RNase E’s CTD and PNPase has allowed us to examine the effects of RNase E upon PNPase. However, our studies are limited by the simplicity of the RNase E-CTD-PNPase assemblies versus the full compositional of BR-bodies in vivo. For example, the full-length RNase E additionally includes the N-terminal endoribonuclease domains and a binding site for the DEAD-box RNA helicase RhlB. This full-degradosome brings the capacity to the breakdown of structured RNA that require RNase E’s endoribonuclease activity followed by the exoribonuclease activity of PNPase. First, we expect the degradation rates of structured RNAs by PNPase alone to be very low. However, adding full-length RNase E and RhlB’s capacity to unwind structured RNA substrates would likely lead to significantly higher rate enhancements.

Secondly, in the context of structured RNA substrates that require a two-step decay process, we suspect that co-localization of RNase E and PNPase is critical to channel the RNA intermediates from RNase E to PNPase and minimize RNA intermediate half-life. In the absence of full-length RNase E localization with PNPase, RNA decay intermediates that RNase E has initially degraded are expected to have a longer half-life before encountering the downstream PNPase enzyme. The addition of the remaining clients (RNase D, Aconitase, and RhlB) will very likely alter some of the protein–protein interactions within the condensate and may introduce new interactions. The full degradosome's altered multivalent interaction networks may have a different chemical environment than the CTD alone. This full degradosome chemical environment may impact viscosity, pH, dielectric constant, increase crowding due to fully populated degradosome, access RNA substrates to the ribonuclease enzymes, and could further refine substrate selectivity. Future comparison of unoccupied RNase E biomolecular condensates to BR-bodies with partial client occupancy to full client occupancy will yield considerable insights into the systems biology of BR-bodies.

From past studies, it is well known that RNase E’s CTD domain regulates the functions of PNPase^32,35^. Here we considered the mechanism of how RNase E regulates the exoribonuclease functions of PNPase. As a part of this work, we identified a PNPase variant that disrupts the RNase E-PNPase interaction, indicating a specific RNase E-PNPase interaction site mediates their recruitment into the RNase E biomolecular condensates. Moreover, this interaction leads to a 3.4-fold enhancement of PNPase’s enzymatic functions and a 5.4-fold enhancement in selectivity for poly(A) over poly(U) substrates (Fig. 4). However, our past in vivo studies in *C. crescentus* indicated that the percent of A ribonucleotides is negatively correlated (R = − 0.52) with BR-body enrichment^27^. This may suggest that long contiguous stretches of adenosine display preferences over isolated adenosine bases amongst oligoribonucleotide substrates.

Alterations of substrate specificity have been a common theme in studying how biomolecular condensates impact enzyme functions. For example, studies from Peeples et al. systematically considered how the SUMOlyation enzymatic cascade was regulated by biomolecular condensates^43^. A key observation of their studies was that changes in enzyme activity were due to scaffold-induced changes in substrate *K*_m_^43^. In comparison, we found that PNPase functioning within RNase E biomolecular condensates displays a considerable substrate preference for degrading poly(A) over poly(U) (Fig. 4a,d). This decreased performance towards the poly(U) substrate is partially rooted in differences in the degree of RNA enrichment (Fig. S5c). In addition, poly(U) may alter the RNase E-PNPase biomolecular condensate viscosity and the potential benefits of mass action. This preference for poly(A) over poly(U) is intriguing, as polyadenylation has been implicated in mRNA degradation in *E. coli*^44,45^ and *C. crescentus*^46^. Indeed, results suggest that BR-bodies can fine-tune the half-lives of RNAs in cells and that polyadenylation may shape the available transcriptome. Therefore, future studies will be needed to examine the interplay of polyadenylation and BR-body functions in vivo and in vitro.

Beyond mass action impacts upon client enzymes, the interaction between scaffold and client may allosterically regulate enzyme functions. Here we showed that the addition of the GWW peptide did not stimulate changes in PNPase function (Fig. 4e). Our current work has not found evidence that allosteric regulation plays a major role in how BR bodies impact PNPase’s enzymatic functions. In contrast, our past studies showed that the PodJ scaffold allosterically regulates the histidine kinase PleC through interaction with PleC’s sensory domain^47^. The coupling of enzyme recruitment and enzyme regulation enabled the spatial regulation of PleC function, which is critical for cell polarity. Our studies suggest that systematically evaluating biomolecular condensate effects through selective scaffolding, mass action, and allostery can reveal how biomolecular condensates fine-tune enzyme functions.

Our studies point indicate the biochemical steps, reactants and products of client enzymes may be logically connected to the material properties of their associated biomolecular condensates. In vivo, RNase E depends upon heterotypic phase separation with RNA substrates^26^. In this study, we showed that the availability of a second critical PNPase reactant, phosphate, also regulates the phase properties of RNase E (Fig. 5). We found that phosphate-poor conditions enhance RNase E’s phase separation in vitro and the number of BR-bodies in vivo (Fig. 5)*.* Changes in the media’s phosphate concentrations may also increase PNPase activity clearing RNAs from cells, leading to BR-body disassembly. Moreover, the faster growth rate of *C. crescentus* in high phosphate would result in more ribosomes competing with BR bodies. Therefore, phosphate nutrient availability regulates BR phase separation and access to RNA substrates. Under low phosphate conditions, in the absence of phosphate, PNPase activity may be too low to drive the decay of RNAs. Moreover, low phosphate conditions in the presence of nucleotide diphosphates may bias PNPase's function as a polymerase. This presents significant evolutionary constraints on microorganisms that live in low phosphate nutrient conditions. This critical phosphate-BR-body feedback loop ensures robust PNPase activity as *Caulobacter crescentus* endures fluctuating availability of phosphate nutrients in the environment.

The mechanism of how phosphates dissolve RNase E condensates will require understanding the RNase E phase separation mechanism. We currently propose that protein–protein interactions between RNase E’s positively and negatively charged blocks play a key role in RNase E’s homotypic phase separation. Whereas in RNA-stimulated phase separation, RNase E’s positively charged block interacts with negatively charged RNA. Therefore, we speculate that the negatively charged phosphates interact with the positively charged blocks in a way that competes with and disrupts key multivalent interactions that drive RNase E’s phase separation. This competition could be direct competition for binding to the same interaction motifs, or it may be indirect, where salts may alter RNase E’s conformation to prevent multivalent interactions. Delineating these models will require understanding the multivalent interaction mediating RNase E phase separation.

More broadly, substrate-mediated feedback on phase separation may be a common way to logically connect phase separation to enzymatic functions. Previous studies from Saurabh et al. showed that the critical substrate of histidine kinases, ATP, could also readily dissolve SpmX biomolecular condensates that regulate histidine kinase biochemistry at concentrations of 2 mM^48^. PNPase shares similarities to this example, as its key substrate (phosphate) also attenuates RNase E phase separation at high concentrations. RNA substrate availability is also required to form BR-bodies in vivo. This collectively suggests that substrate-mediated positive and negative feedback loops may commonly regulate phase separation in cells. Interestingly, both RNase E and SpmX biomolecular condensates are sensitive to phosphate nutrient availability. RNase E phase separation is directly sensitive to phosphate levels, whereas the SpmX condensates are sensitive to cellular ATP levels. These studies highlight the intricate logical wiring of biomolecular condensate biochemistry and material properties with nutrient availability.

## Methods

### Protein expression and purification

#### Purification of PNPase-mCherry

Plasmid pMJC0095 was constructed to express PNPase from *C. crescentus* with an N-terminal 6x-His-tag and a C-terminal mCherry. Plasmid pTEV5-PNPase-mCherry was transformed into chemically competent Rosetta (DE3) cells and plated onto LB-Miller plates supplemented with 50 mg/mL chloramphenicol, 100 mg/mL ampicillin and incubated overnight at 37 °C. From a single colony, an overnight 60 mL LB-Miller culture (30 mg/mL chloramphenicol, 50 mg/mL ampicillin) was inoculated and incubated at 37 °C. From this saturated culture, 6 L of LB-Miller media (30 mg/mL chloramphenicol, 50 mg/mL ampicillin) was inoculated with 6 mL of the saturated culture and grown to mid-log phase (~ 0.5 OD600). Expression of PNPase-mCherry was induced with 333 µM isopropyl-β-d-1-thiogalactopyranoside (IPTG) for 4 h at 25 °C. The cells were collected by centrifugation at 4 °C, 4000*g*, for 30 min. The resulting pellet was washed with 60 mL resuspension buffer (50 mM Tris pH 7.5, 500 mM NaCl) before being pelleted again at 4 °C, 4000*g*, for 20 min and stored at − 80 °C.

The cell pellet was thawed on ice and then resuspended in 10 mL lysis buffer per liter of culture (20 mM Tris HCl pH 7.5, 500 mM NaCl, 5 mM imidazole, 200 U benzonase) supplemented with SigmaFast protease inhibitor tablets (Sigma). The cell suspension was lysed by continuous passage through an Avestin Emulsiflex-C3 at 15,000 psi for 15 min at 4 °C. Cell debris was pelleted by centrifugation at 29,000*g* for 45 min at 4 °C. The supernatant was loaded onto a HisTrap FF column (GE Healthcare) and washed with 20 column volumes of wash buffer (20 mM Tris HCl pH 7.5, 500 mM NaCl, 5 mM imidazole). Then was eluted with elution buffer (20 mM Tris HCl pH 7.5, 500 mM NaCl, 500 mM imidazole). Fractions containing PNPase were supplemented with 50 mM sodium phosphate (pH 7.5) at 37 °C for 1 h to drive phosphorolysis of co-purifying RNA. The fractions were loaded onto a G-Sep™ 6–600 kDa Size Exclusion Columns (G-Biosciences) and eluted with storage buffer (20 mM Tris HCl pH 7.5, 500 mM NaCl, 5% (v/v) glycerol). Fractions containing PNPase were concentrated using 50,000 MWCO Amicon centrifugal filters to 15.5 mg/mL, aliquoted, and flash-frozen in liquid nitrogen and stored at − 80 °C.

#### Purification of RNase E-CTD-YFP

RNase E CTD was purified as described previously^26^, and summarized here. Cells were thawed on ice and then resuspended in 10 mL lysis buffer per liter of culture (20 mM Tris HCl pH 7.5, 1 mM NaCl, 20 mM imidazole, 1 mM beta-mercaptoethanol, 20 U DNase I, 100 U RNaseA, 200 U benzonase, 0.1% Triton X-100) supplemented with SigmaFast protease inhibitor tablets (Sigma). The cell suspension was lysed by continuous passage through an Avestin Emulsiflex-C3 at 15,000 psi for 15 min at 4 °C. Cell debris was pelleted by centrifugation at 29,000*g* for 45 min at 4 °C. The supernatant was loaded onto a HisTrap FF column (GE Healthcare) and washed with 20 column volumes of wash buffer (20 mM Tris HCl pH 7.5, 1000 mM NaCl, 20 mM imidazole, 1 mM beta-mercaptoethanol). Subsequently, the purification was eluted with elution buffer (20 mM Tris HCl pH 7.5, 1000 mM NaCl, 500 mM imidazole, and 1 mM beta-mercaptoethanol). Fractions containing RNase E were loaded onto a G-Sep™ 6–600 kDa Size Exclusion Columns (G-Biosciences) and eluted with storage buffer (20 mM Tris HCl pH 7.5, 500 mM NaCl, 1 mM beta-mercaptoethanol). Fractions containing RNase E were concentrated using 50,000 MWCO Amicon centrifugal filters to 18.6 mg/mL, aliquoted, flash-frozen in liquid nitrogen and stored at − 80 °C.

MBP-RNase E-CTD-eYFP was purified similarly to RNase E-CTD-eYFP through the nickel affinity chromatography step. After MBP-RNase E-CTD-eYFP was eluted from the HisTrap column, the protein was desalted using a PD-10 column (GE healthcare) with heparin column binding buffer (20 mM Tris HCl pH 7.5, 1 mM beta-mercaptoethanol, 10% glycerol) before passage over a heparin column (Cytiva). MBP-RNase E-CTD-eYFP was eluted with heparin elution buffer (20 mM Tris HCl pH 7.5, 1 mM beta-mercaptoethanol, 10% glycerol, 2 M NaCl) using a linear gradient. Protein was then buffer exchanged into storage buffer (20 mM Tris HCl pH 7.5, 200 mM NaCl, 1 mM beta-mercaptoethanol, 10% glycerol) using a PD-10 column, concentrated using 50,000 MWCO Amicon centrifugal filters to 23.5 mg/mL, aliquoted, and flash-frozen in liquid nitrogen before storage at − 80 °C.

### Microscopy

Fluorescence microscopy samples were prepared by thawing requisite proteins on ice and mixing a buffer to create a final concentration of 20 mM Tris pH 7.5, 1 mM MgCl_2_, 10 mM Na_2_HPO_4_ pH 7.5, 100 mM NaCl, 10% PEG 8000 (Figs. 2, S1, S2 [120 mM NaCl], S3) or 20 mM Tris pH 7.5, 20 mM MgCl_2_, 4 mM Na_2_HPO_4_ pH 7.5, 70 mM NaCl, 0.5 mM DTT (Figs. 3, 4, 5, S4, S5), to which was added protein and poly(A) at the necessary concentrations specified in each experiment. Imaging samples were pipetted into a 1 mm well formed by an adhesive spacer (Electron Microscopy Sciences) affixed to a microscope slide (VWR) and sealed with a glass coverslip (VWR). Slides were inverted and allowed to sit at room temperature for 30 min before imaging on a Nikon Eclipse Ti-E inverted microscope with a Plan Apo-(lambda) 100×/1.45 oil objective and 518F immersion oil (Zeiss). Excitation filter cubes CFP/YFP/mChy (77074157) and Cy5 (77074160) from Chroma and emission filter sets CFP/YFP/mChy (77074158), and Cy5 (77074161) from Chroma were used for fluorescence imaging with a Spectra X light engine from Lumencor. Images were taken with an Andor Ixon Ultra 897 EMCCD camera.

Images were analyzed with Fiji using a gaussian blur image subtraction and Renyi Entropy threshold method to find droplet boundaries. The mean signal from each droplet area was divided by the mean signal of the non-droplet area to give a partitioning ratio for each droplet, which is averaged to give a partitioning ratio for each experimental condition. Droplet circularity was measured in Fiji using the formula circularity = 4 * π * (area/perimeter^2^).

### Effect of phosphate on BR-bodies in vivo

JS 51 strain (RNE::RNE YFP) was grown in HIGG medium supplemented with either 30 µM or 1 mM phosphate. HIGG medium was prepared as described previously^49^. Using a Nikon NIE fluorescence microscope, the cells were imaged in log phase when the OD reached 0.3–0.4. The acquired images were analyzed using the microbe J plugin in Image J software^50^. For the foci detection in the cells, the tolerance and Z-score were set to 160 and 18, respectively.

### Ionic strength titration

Investigation of the effect of salt concentration on the formation of RNase E condensates was carried out by titrating NaCl, NaH_2_PO_4_/Na_2_HPO_4_ (pH 7.5), Na_2_SO_4_, KCl, KH_2_PO_4_/K_2_HPO_4_ (pH 7.5), and K_2_SO_4_ into RNase E condensate solutions while controlling for ionic strength following the methods detailed by Patel et al*.*^51^ and reproduced here, in brief.

The ionic strength of solutions was calculated using the following equation:

$$I= \frac{i}{2} \sum \limits_{i=1}^{n}{c}_{i}{z}_{i}^{2}$$

where “I” is ionic strength, “i” is the number of ions formed by the salt, “c” is the concentration, and “z” is the charge of the ion. The concentrations of individual salts were adjusted to match the ionic strength of a titration of sodium phosphate at 2, 4, 6, 8, and 10 mM. In addition to the concentration of phosphate or sulfate salts listed in Fig. S7, 50 mM NaCl was included in all NaH_2_PO_4_/Na_2_HPO_4_ (pH 7.5) and Na_2_SO_4_ solutions and 50 mM KCl was included in all KH_2_PO_4_/K_2_HPO_4_ (pH 7.5), and K_2_SO_4_ solutions. Therefore, the formation of RNase E biomolecular condensates at each salt concentration could be compared to the consistent ionic strength of the overall solution. The ionic strength of each solution is listed at the top of Fig. S7, while the concentrations of each salt added to reach the overall ionic strength of the solution are shown underneath each image.

### Turbidity measurements

Turbidity reaction mixtures contained 20 mM Tris pH 7.5, 70 mM NaCl, 0.5 mM DTT, and 20 µM RNase E. The magnesium titration trial contained 4 mM PO_4_ pH 7.5, and the phosphate titration trial contained 20 mM MgCl_2_. The absorbance at 340 nm of triplicate samples was recorded with a Tecan M200Pro microplate reader (Tecan Group Ltd.). A background sample containing only buffer was collected and subtracted from each sample.

### Generation of fluorescent nucleotides

Fluorescent polynucleotides were generated using 5 µM PNPase with 99 µM NDP and 1 mM ADP-Cy5 in a buffer of 20 mM Tris pH 7.5, 100 mM NaCl, 10 mM MgCl_2_, 0.5 mM DTT. Reactions were run for 2 h at room temperature. Fluorescent polynucleotides were purified using silica gel spin columns and frozen at − 80 °C for future use.

### PNPase-mediated RNA-degradation assay

RNA degradation assays were performed at room temperature in 20 mM Tris–HCl (pH 7.5), 70 mM NaCl, 20 mM MgCl_2_, 4 mM Na_2_HPO_4_ (pH 7.5), and 0.5 mM DTT with 5 µM purified PNPase and 20 µM purified RNase E. Reactions were initiated by adding 25 ng/µL poly(A) RNA. For time-course assays, aliquots were withdrawn and quenched in 100 mM EDTA. Samples were denatured in 1.5 volumes of 2 × RNA loading buffer containing 95% formamide, 18 mM EDTA, and 0.025% SDS and incubated at 95 °C for 3 min. Quenched ribonuclease reaction aliquots were loaded onto a pre-run 6% acrylamide gel containing 7 M urea. The gel was run in 1 × TBE (89 mM Tris base, 2 mM EDTA, 89 mM boric acid) at 250 V at room temperature to separate RNA. Subsequently, the gel was rinsed in Milli-Q water for 5 min and stained for 20 min with 1 × SYBR gold nucleic acid stain (Invitrogen) in 1 × TBE. Each gel assay included RNA-only and protein-only controls. Gels were imaged with BioRad ChemiDoc™ MP imager using SYBR gold settings and quantified using the BioRad ImageLab software package. The intensity of the protein-only lane was subtracted from each timepoint lane intensity and plotted against time. The degradation rate was calculated in relation to a known amount of RNA added in an RNA-only control lane and divided by the amount of protein to give rates in µg min^−1^ (mg PNPase)^−1^. Since poly(A) is of heterogenous length, nuclease activity cannot be reported on a molar scale.

### PNPase-mediated RNA-degradation assay with GWW peptide

The 10 C-terminal residues of *Caulobacter crescentus* RNase E (EKPRRGWWRR) (GWW peptide) were synthesized by GenScript with C-terminal amidation and dissolved in Milli-Q water and frozen at − 80 °C until further use. In RNA-degradation assays containing GWW peptide, the peptide was added to the reaction tube with buffer and PNPase and incubated at room temperature for 30 min to allow the peptide to associate with PNPase. Reactions were otherwise carried out as described previously.

### Statistical analysis

Graph-pad Prism 9 software for Mac was used for statistical analysis. The analysis of variance (ANOVA) test was used to evaluate the significant differences between conditions, where a *p* ≤ 0.05 was considered significant. Data for enzyme rate analysis represents the mean and standard deviation of three replicates. Data for partition ratio analysis represents the mean and standard deviation of at least 18 images taken from at least three replicates. Tukey’s multiple comparisons test was used to determine significant differences between groups.

### Supplementary Information

Supplementary Information.

## Data Availability

The datasets used and/or analyzed in this study are available from the corresponding author on reasonable request.
