# Supplementary material for: RNase E biomolecular condensates stimulate PNPase activity
Source: Sci Rep. 2023 Aug 9;13:12937. doi: 10.1038/s41598-023-39565-w (PMC10412687; doi:10.1038/s41598-023-39565-w)
Supplement: Supplementary file 1 — Supplementary Information. [file 41598_2023_39565_MOESM1_ESM.pdf]

Supporting Information:

RNase E biomolecular condensates stimulate PNPase activity

Michael J. Collins[1], Dylan T. Tomares[1], Vidhyadhar Nandana[2], Jared M. Schrader[2], W. Seth Childers[1]

[1] University of Pittsburgh Department of Chemistry. [2] Department of Biological Sciences, Wayne State University, Detroit, MI 48202, USA

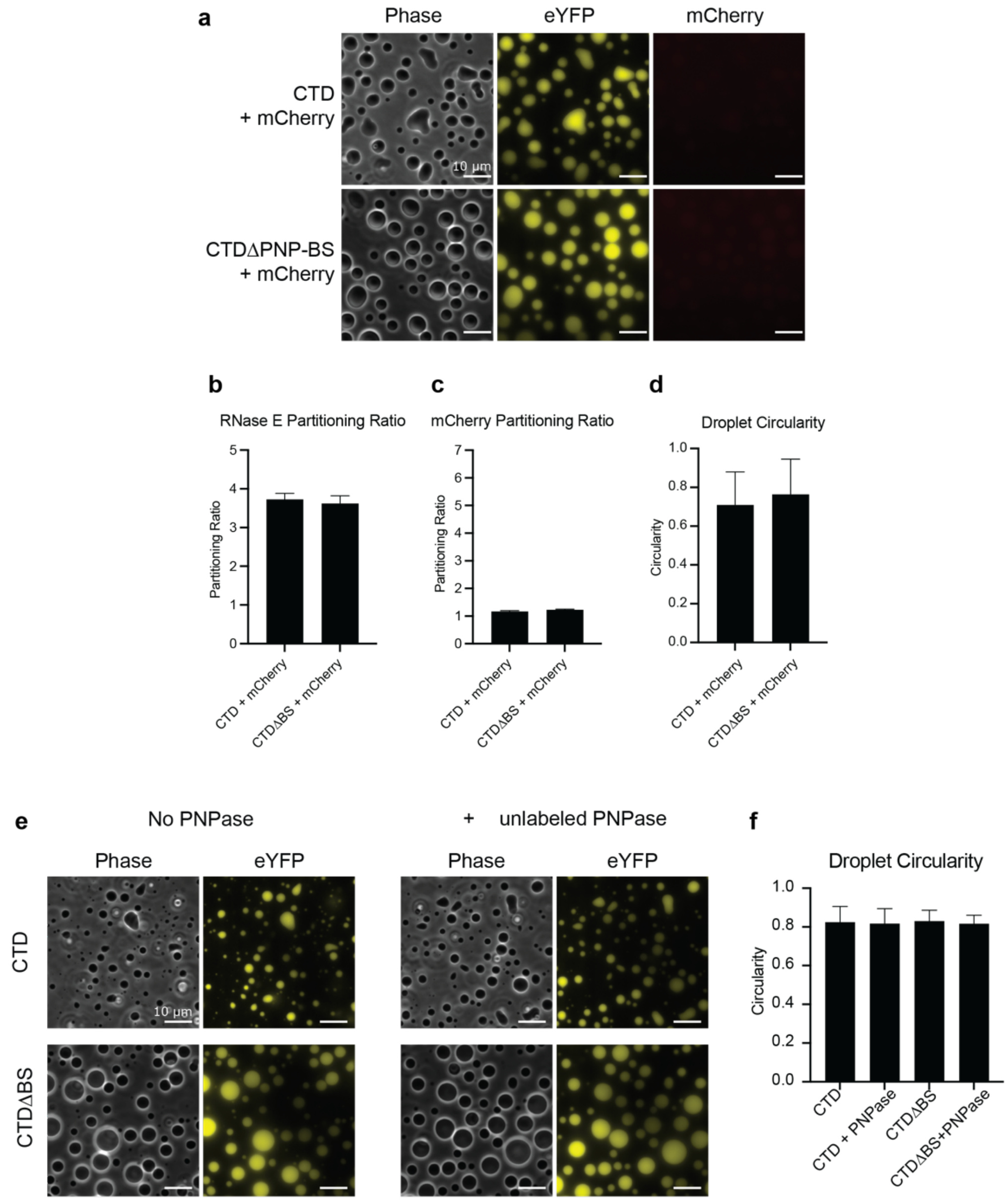

Figure S1: Weak fluorescent protein interactions between YFP and mCherry do not lead to the recruitment of mCherry into RNase E CTD-YFP or RNase E CTDΔPNP-BS protein-rich biomolecular condensates. (a) Phase contrast and fluorescence microscopy images of 20 μM RNase E biomolecular condensates mixed with 1 μM mCherry. RNase E and RNase E-ΔPNP-BS contain a C-terminal YFP tag. The scale bar is 10 μm. (b) Average partitioning ratios and standard deviations are presented for RNase E CTD-YFP and RNase E CTDΔPNP-BS-eYFP. There is no significant difference between PRs ($p > 0.05$). (c) Average partitioning ratios and

standard deviations are presented for mCherry. There is no significant difference between PRs ($p > 0.05$). Data represent average and standard deviations of n > 300 droplets. (d) Droplet circularity measurements are presented as the average and standard deviation of n > 300 droplets. See the methods section for the droplet circularity calculation formula. (e) Microscopy images of RNase E- CTD-YFP and RNase E CTD∆PNP BS-YFP show that the addition of unlabeled PNPase does not affect the morphology of RNase E. (f) Circularity measurements of droplets before and after the addition of unlabeled PNPase show that PNPase does not significantly alter the circularity of droplets formed from 20 µM RNase E CTD-YFP or 20 µM RNase E CTD∆PNP BS-YFP. The average and standard deviation of circularity measurements for n > 300 droplets are shown.

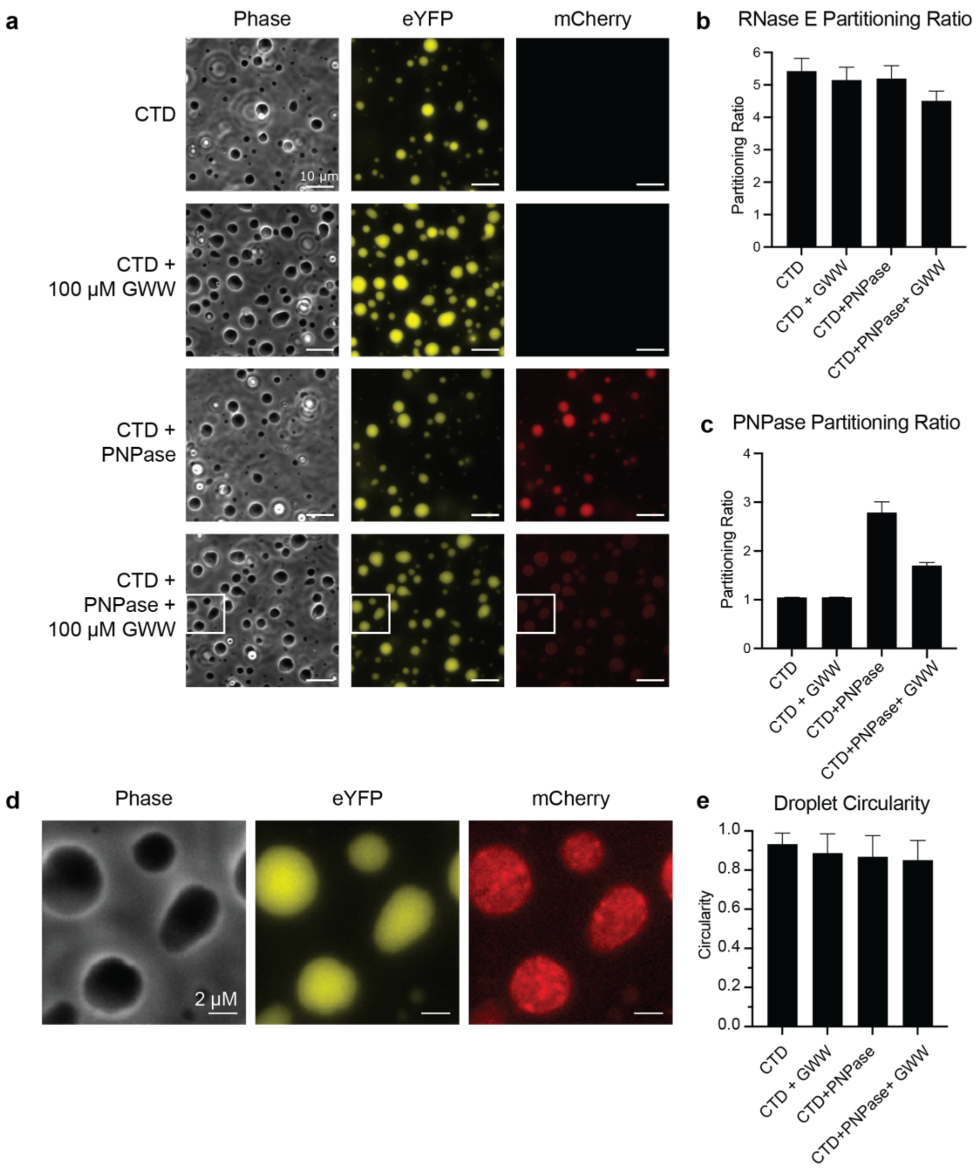

Figure S2: The RNase E GWW peptide can chase PNPase-ASM-mCherry out of RNase E biomolecular condensates. (a) Phase contrast and fluorescence microscopy images of protein-rich biomolecular condensates formed by RNase E CTD-YFP were incubated with a peptide comprised of the 14 C-terminal residues of RNase E (called the GWW peptide). A mixture of 1 µM PNPase together with 100 µM of the GWW peptide was incubated for 60 minutes. After this incubation, the mixture was added to 20 µM of RNase E biomolecular condensates. Buffer conditions were 20 mM Tris pH 7.5, 120 mM NaCl, 1 mM $MgCl_2$, 10 mM $NaPO_4$ pH 7.5, 10%

PEG. Partitioning coefficients of RNase E CTD-YFP (b) and PNPase-ASM-mCherry (c) from three replicates containing over 300 droplets. The lower partitioning ratio for PNPase is likely due to the 120 mM NaCl concentration which is higher than was used in other imaging experiments. GWW peptide does not significantly alter RNase E droplet formation or partitioning ratios. However, it does significantly decrease the partitioning of PNPase into the droplets. (d) Zoomed images of the white boxes in panel (a) showing the patchy localization of PNPase in the RNase E condensate. The scale bar is 2 μm. (e) Droplet circularity measurements of RNase E or RNase E and PNPase with and without GWW peptide represent the average and standard deviation of $n > 300$ droplets.

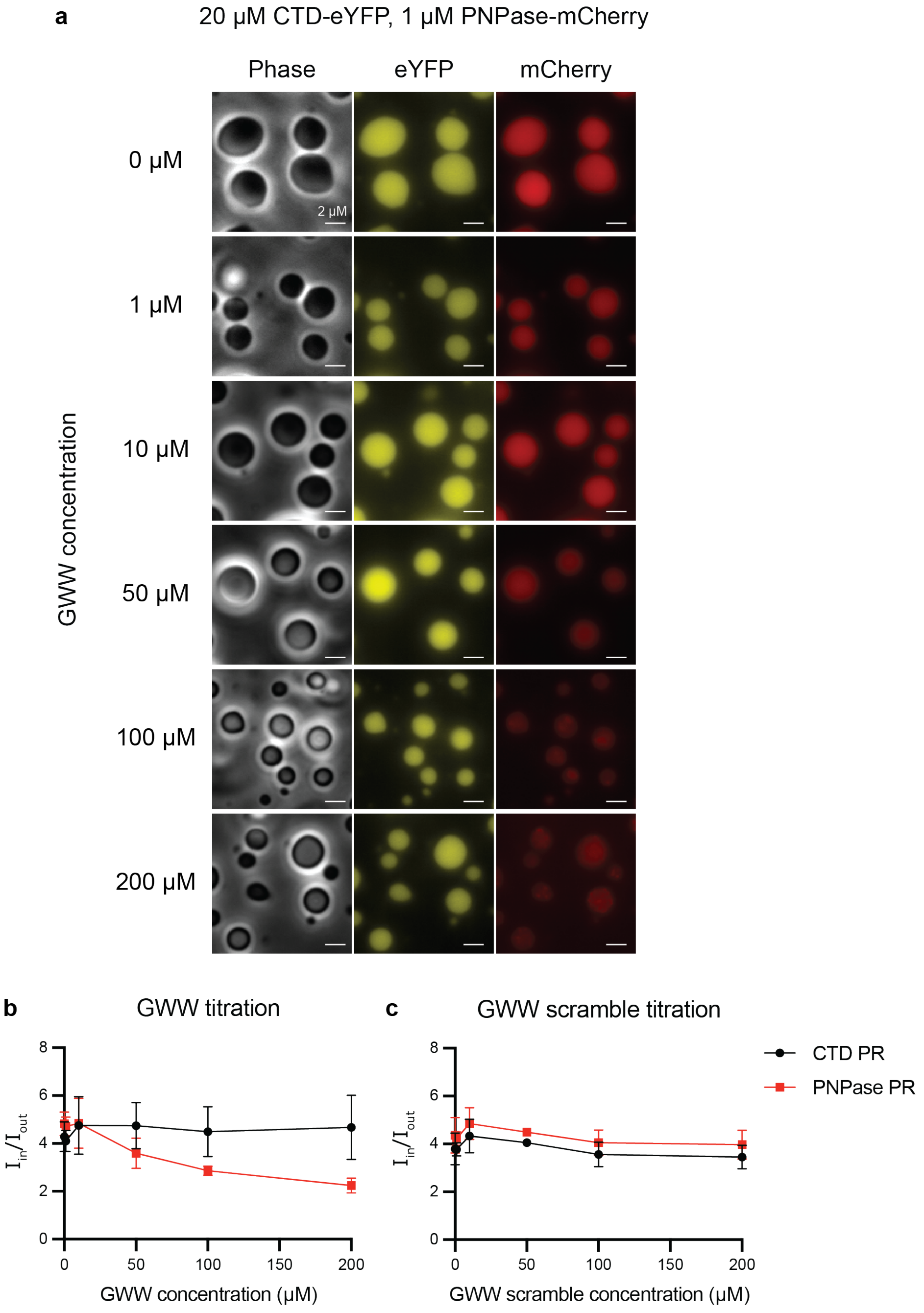

Figure S3: Incubation of the GWW peptide with PNPase before addition to RNase E condensates results in a patchy appearance of PNPase at or above 100 μM GWW peptide. (a) Microscopy images of each point in the GWW titration show the patchy localization of PNPase

in the RNase E condensate at and above 100 μM GWW peptide. The scale bar is 2 μm. (b) A titration of GWW peptide shows the dose-dependent inhibition of PNPase recruitment into RNase E droplets. Black line, RNase E partitioning ratio; Red line, PNPase partitioning ratio. (c) A titration of the GWW scrambled peptide does not show an inhibition of PNPase partitioning into RNase E droplets. GWW or GWW scramble was titrated into 20 μM RNase E CTD-YFP and 1 μM PNPase-mCherry. Partitioning ratio data shown are an average and standard deviation of three trials representing over 300 droplets.

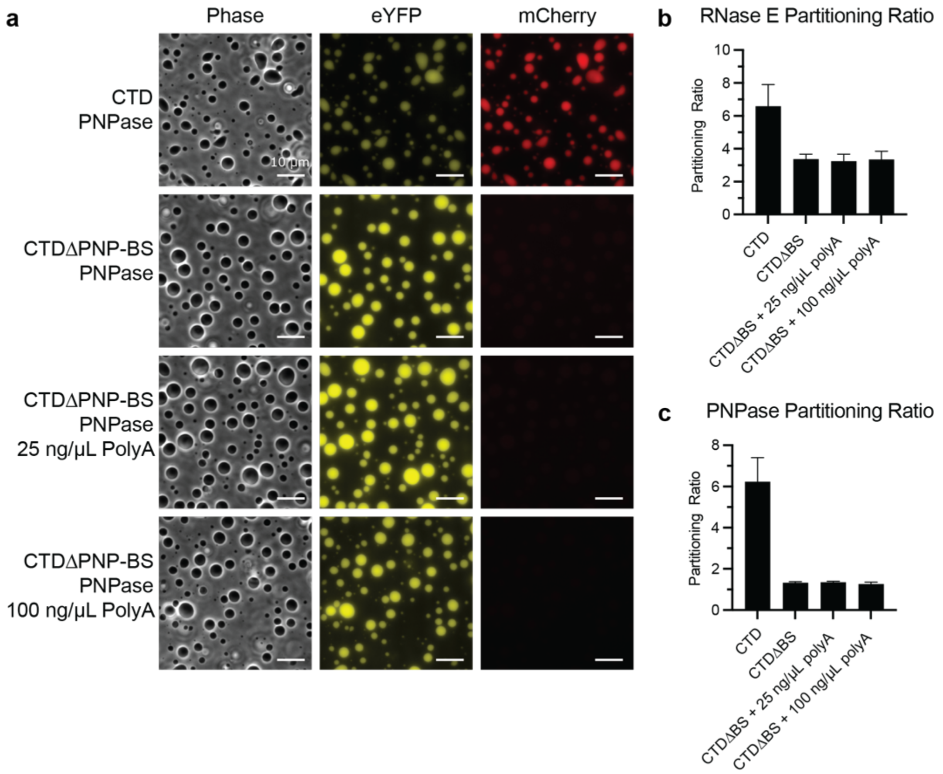

Figure S4: Poly(A) RNA is insufficient to shepherd PNPase into condensates formed by RNase E-ΔBS. (a) Phase contrast and fluorescence microscopy images of RNase E-rich or RNase E-ΔBS-rich biomolecular condensates were incubated with 5 μM of PNPase-ASM-mCherry. 25 or 100 ng/μL poly(A) RNA added to droplets formed with RNase E-ΔBS did not show incorporation of PNPase. The average and standard deviation of partitioning coefficients of RNase E (b) and PNPase (c) from over 300 droplets show a significant decrease in PNPase partitioning into condensates formed with RNase E-ΔBS even in the presence of poly(A) RNA.

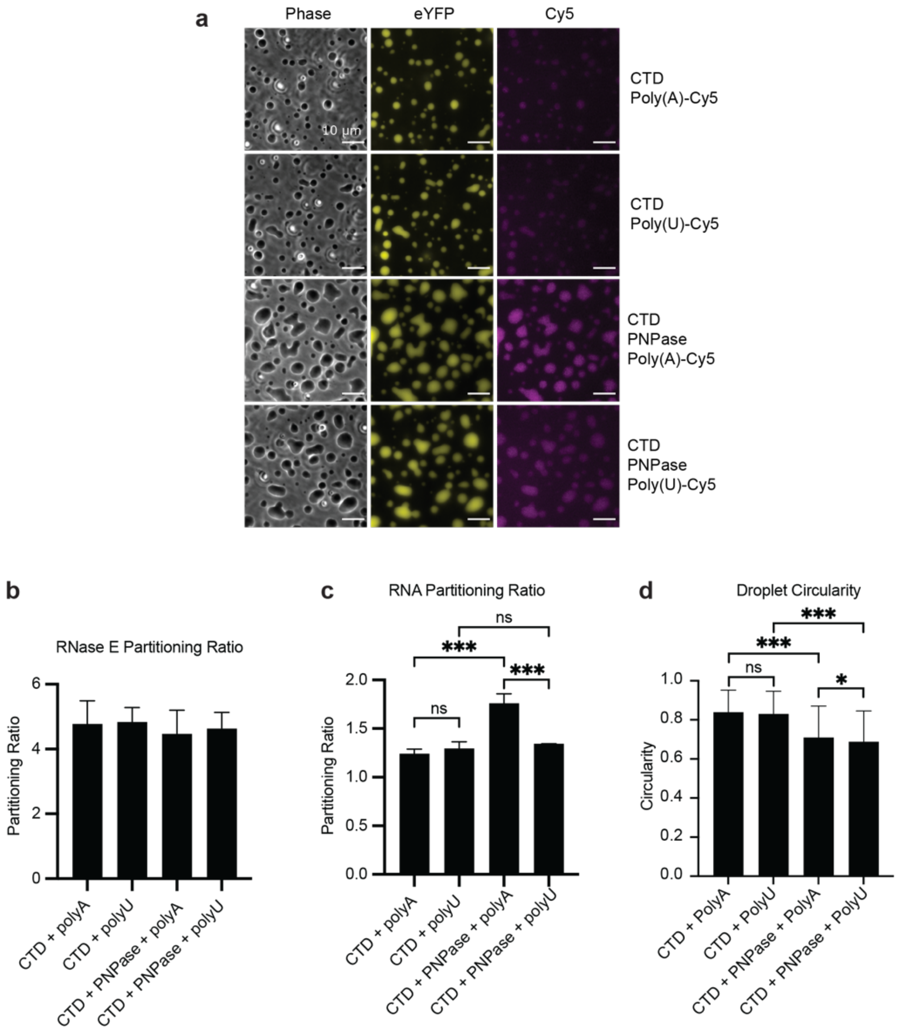

Figure S5: Poly(U) is poorly enriched in RNase E biomolecular condensates. (a) Phase contrast and fluorescence microscopy images of 20 µM of RNase E CTD-YFP with 25 ng/µL Cy5-labeled RNA homopolymers poly(A) or poly(U) (top two rows of images) or 20 µM of RNase E CTD-YFP and 5 µM PNPase-ASM-mCherry with 25 ng/µL Cy5-labeled RNA homopolymers poly(A) or poly(U) (bottom two rows of images). Labeled RNA consists of 10 ng/µL Cy5-labeled RNA mixed with 15 ng/µL unlabeled RNA. Scalebar = 10 µm. (b) Partitioning ratios for RNase E are calculated as the average and standard deviation of n > 300 droplets. (c) Partitioning ratios for Cy5-labeled poly(A) and poly(U) are calculated as the average and standard deviation of n > 300 droplets. (d) Droplet circularity of RNase E condensates with Cy5-labeled poly(A) and poly(U) represent the average and standard deviation of n > 300 droplets.

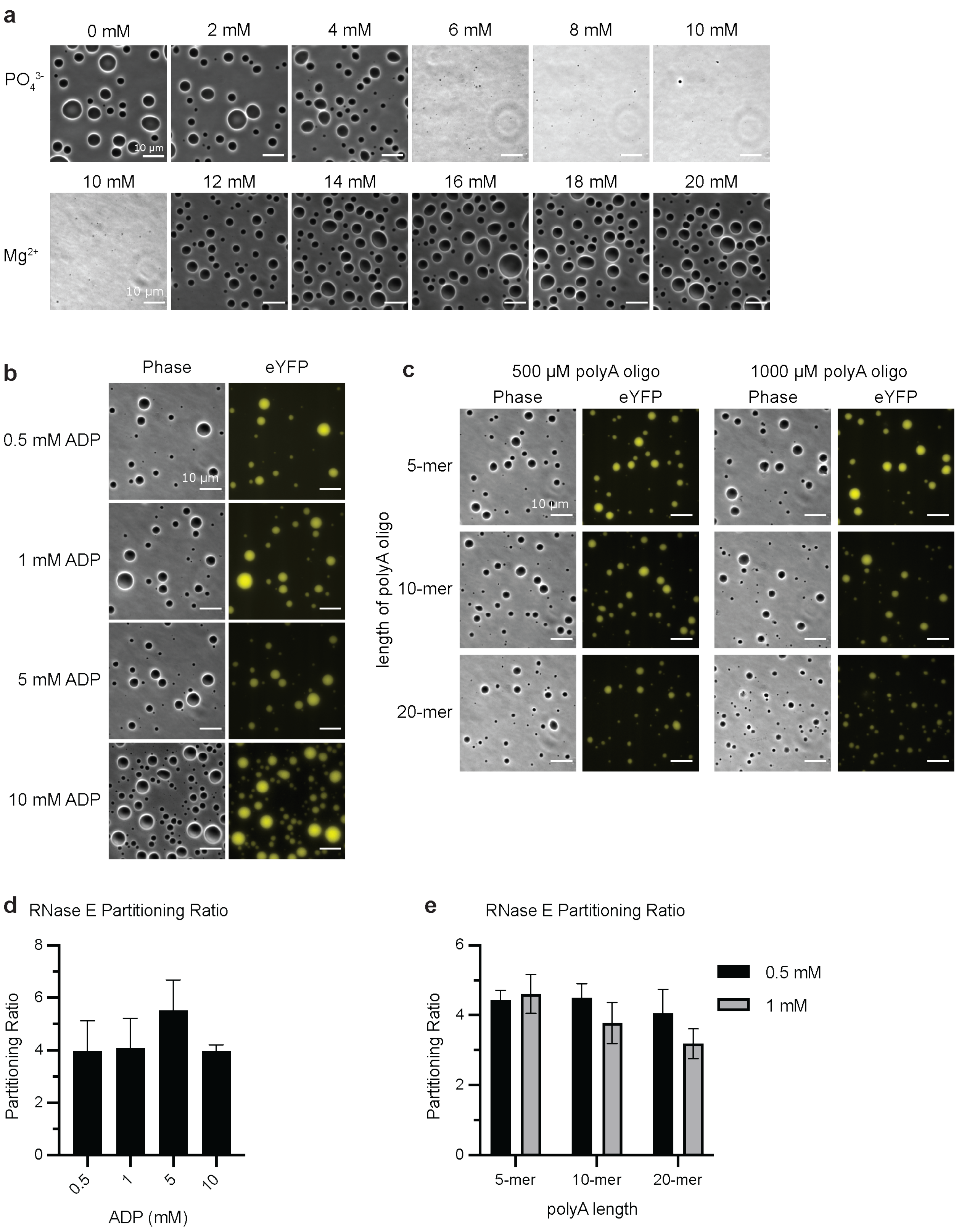
a
0 mM
2 mM
4 mM
6 mM
8 mM
10 mM
PO4 3-
10 μm
10 mM
12 mM
14 mM
16 mM
18 mM
20 mM
Mg2+
10 μm
b
Phase
eYFP
0.5 mM ADP
10 μm
1 mM ADP
5 mM ADP
10 mM ADP
c
500 μM polyA oligo
1000 μM polyA oligo
Phase
eYFP
Phase
eYFP
length of polyA oligo
5-mer
10 μm
10-mer
20-mer
d
RNase E Partitioning Ratio
Partitioning Ratio
8
6
4
2
0
0.5
1
5
10
ADP (mM)
e
RNase E Partitioning Ratio
Partitioning Ratio
6
4
2
0
0.5 mM
1 mM
5-mer
10-mer
20-mer
polyA length

Figure S6: Sodium phosphate and magnesium chloride regulate the formation of RNase E-CTD biomolecular condensates. (a) Phase contrast images of 20 µM of RNase E in 20 mM $MgCl_2$ mixed with 0-10 mM sodium phosphate (top row). Phase contrast images of 20 µM of RNase E in 4 mM sodium phosphate mixed with 0-20 mM magnesium chloride (bottom row). (b) ADP at 0-10 mM concentrations was added to a solution of 20 µM of RNase E CTD-YFP and subsequently imaged by phase contrast and fluorescence microscopy. (c) Short poly(A) oligoribonucleotides (5-mer, 10-mer, or 20-mer) were added to a solution of 20 µM of RNase E CTD-YFP and subsequently imaged by phase contrast and fluorescence microscopy. Scalebar = 10 µm. (d) Partitioning coefficients of RNase E CTD-YFP during ADP titration. (e) Partitioning coefficients of RNase E CTD-YFP with short poly(A) oligomers. Partitioning coefficients represent the average and standard deviation of n > 150 droplets.

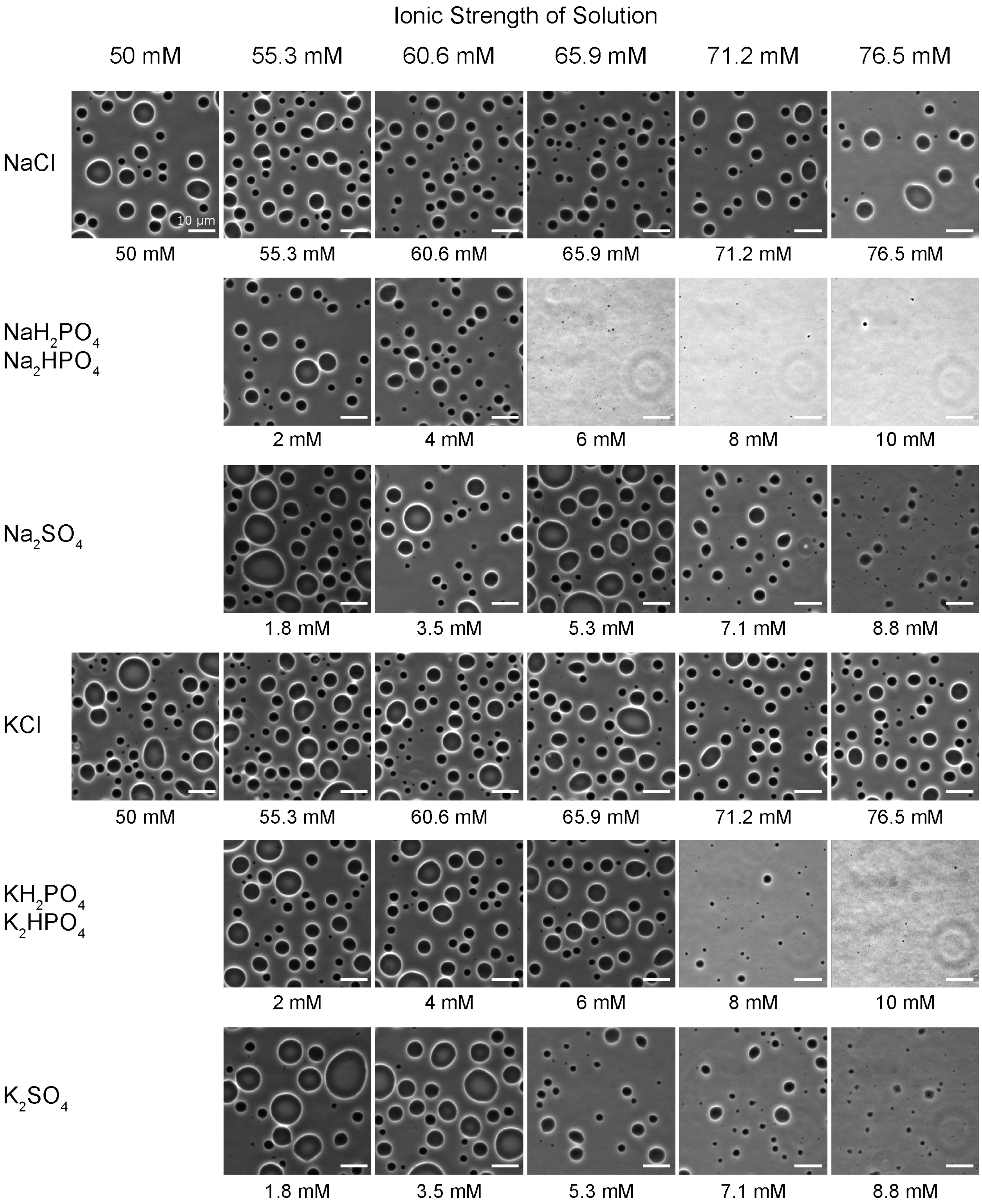

Figure S7: Phase contrast images of titration series of sodium chloride, sodium phosphate, and sodium sulfate into a 20 μM RNase E (unlabeled) solution. The sodium phosphate and sodium

sulfate titration series also contains 50 mM NaCl. Images are organized in columns according to their ionic strength, listed in mM at the top of the figure. Concentrations of chloride and sulfate salts were adjusted to match the ionic strength of the phosphate titration. The buffer includes 20 mM Tris pH 7.5, 20 mM $MgCl_2$, 0.5 mM DTT, 20 μM unlabeled RNase E, and 50 mM NaCl except in the NaCl titration, where the NaCl concentration is listed below the image. Concentrations.

Supplemental Materials, Methods, and Procedures

Plasmids, primers, and strains

Table 1. Plasmids used in this study.

Plasmid	Description	Reference
pTEV5	Bacterial expression vector	[1]
pMJC0094	His6x-PNPase	This study
pMJC0095	His6x-PNPase-mCherry	This study
pMJC0112	His6x-PNPase-S439A/S440A/S441A active site mutant	This study
pMJC0113	His6x-PNPase-S439A/S440A/S441A-mCherry active site mutant	This study
pMJC0119	His6x-RNase E(CTD)-Δ884-898-eYFP PNPase binding site deletion	This study
pMJC0137	His6x-PNPase-V104A/E224A/F233A-mCherry	This study
pDT177	His6x-pTEV5-RNase E CTD-YFP	This study
pDT279	His6x-pTEV5-MBP-RNase E CTD-YFP	This study

Table 2. Strains used in this study.

Strain	Description	Reference
E. coli DH5α	Bacterial cloning strain	Invitrogen
E. coli Rosetta (DE3) pLysS	Bacterial expression strain	Novagen
MJC192	Rosetta-pMJC0094 PNPase expression strain	This study
MJC196	Rosetta-pMJC0095 PNPase-mCherry expression strain	This study
MJC212	Rosetta-pMJC0112 PNPase-S439-441A expression strain	This study
MJC213	Rosetta-pMJC0113 PNPase-S439-441A expression strain	This study

MJC235	Rosetta-pMJC0119 RNase E CTDΔPNPase_BS-eYFP expression strain	This study
MJC303	Rosetta-pMJC0137 PNPase-V104A/E224A/F233A expression strain	This study
DTT210	Rosetta-pDT279 MBP-RNase E CTD-YFP expression strain	This study
JS51	NA1000 RNE::RNE-YFP $Spec^R$	[2]

Table 3. Primers used in this study.

Primer	5'-3' sequence	Description
MJC222	GAAAACCTGTATTTTCAGGGCGCTATGTTCGAT ATCAAACGCAAGACGATCGAGTGG	PNPase forward
MJC223	GCTCGAGAATTCCATGGCCATATGGCTTTACGC CTCTTCGGCCGCCG	PNPase reverse
MJC224	CTCGAGATCTTAACGCCTCTTCGGCCGCCGCTT CC	PNPase-mCherry reverse
MJC225	GGCCGAAGAGGCGTTAAGATCTCGAGCTCCGG AGAATTCG	mCherry forward
MJC226	GCTCGAGAATTCCATGGCCATATGGCTTTACTT GTACAGCTCGTCCATGCCGCC	mCherry reverse
MJC261	AGACCGTGGCCATCGCCGCCGCACCGTTGCTC TCGGTGATCTCCG	PNPase 1-438 (S439-441A) active site mutant reverse
MJC262	CGGTGCGGCGGCGATGGCCACGGTCTGCGGT TCG	PNPase 442-713 active site mutant forward

MJC265	CCAACTAGTGAAAACCTGTATTTTCAGGGCGCT ATGACCGGCGTGCTGGAAGG	RNase E(CTD) forward
MJC269	GCTCGAGAATTCCATGGCCATATGGCTTTACTT GTACAGCTCGTCCATGCCCA	EYFP reverse
MJC280	CGGAGCTCGAGATCTTAAGATCTCGTTCGGATC CGGCTCG	RNE-ΔPNP_BS reverse
MJC281	GGATCCGAACGAGATCTTAAGATCTCGAGCTCC GGAGAATTCG	EYFP forward
MJC282	TGTCTTCCGGCTCCGCCGCGAAGGGCTCCTTG GCGG	PNPase-F233A reverse
MJC283	GCCCTTCGCGGCGGAGCCGGAAGACACCGAC GCG	PNPase-F233A forward
MJC290	TCTTGAAGCCCTTCGCGAACAGCGGGCGGATC GGAC	PNPase-V104A reverse
MJC291	CCCGCTGTTCGCGAAGGGCTTCAAGAACGAAG TC	PNPase-V104A forward
MJC292	GCGGCGTGCGCGGCCAGGTCGATGATCGCGT CG	PNPase-E224A reverse
MJC293	ATCATCGACCTGGCCGCGCACGCCGCCAAGGA GCCCTTCG	PNPase-E224A forward
DT471	TTTTCAGGGCGCTAAAATCGAAGAAGGTAAACT GGTAATCTGG	MBP-forward
DT473	GCACGCCGGTCATACCTTGGAAGTAGAGATTCT CTGACGTGG	MBP-reverse

DT476	CTACTTCCAAGGTATGACCGGCGTGCTGGAAG G	RNase E(CTD)-YFP forward
DT477	TGGCCATATGGCTTTACTTGTACAGCTCGTCCA TGCCG	RNase E(CTD)-YFP reverse

Plasmid construction

Plasmid pMJC0094, PNPase expression vector: pMJC0094 was generated in pTEV5 using the Gibson DNA assembly method [1, 3]. Primers for Gibson reactions were designed using J5 DNA assembly design automation software [4]. The resulting plasmid encodes a hexahistidine tag, followed by a TEV protease cleavage site at the N-terminus of PNPase. Full-length *C. crescentus* PNPase encoding residues 1-713 was amplified by PCR using genomic DNA of *Caulobacter crescentus* NA1000 as a template with primers MJC222 and MJC223. These primers include overhang sequences homologous to regions flanking the NheI site in pTEV5. Vector pTEV5 was linearized by digestion with NheI restriction enzyme and ligated with PCR fragments following the Gibson method described here briefly. Equimolar amounts of linearized pTEV5 and PNPase PCR fragments were incubated at 50 °C for 60 min in a reaction mixture containing 5% PEG-8000, 100 mM Tris-HCl pH 7.5, 10 mM $MgCl_2$, 10 mM DTT, 0.2 mM of each of the four dNTPs, 1.25 mM NAD, 5.3 mU/µL T5 exonuclease, 33.3 mU/µL Phusion DNA polymerase, and 5.3 U/µL Taq ligase (NEB) resulting in plasmid pMJC0094 that encodes an N-terminal His_6-tag and TEV cleavage site (MSYYHHHHHHDYDIPTSENLYFQGAM) fused to PNPase.

Plasmid pMJC0095, PNPase-mCherry expression vector: pMJC0095 encoding PNPase with a C-terminal flexible linker and mCherry was generated in pTEV5 using the Gibson assembly method. PCR fragments encoding full-length PNPase were cloned from pMJC0094 using primers MJC222 and MJC224. A PCR fragment encoding a linker (LRSRAPENSNVTRHRSAT) and mCherry was cloned from pDT027 using primers MJC225 and

MJC226. Primers MJC222 and MJC226 include overhang sequences homologous to regions flanking the NheI site in pTEV5. Gibson assembly was completed as previously described resulting in plasmid pMJC0095 that encodes an N-terminal His_6-tag and TEV cleavage site (MSYYHHHHHHDYDIPTSENLYFQGAM) fused to PNPase with a C-terminal flexible linker (LRSRAPENSNVTRHRSAT) and mCherry.

Plasmid pMJC0112, PNPase-mCherry active site mutant expression vector: pMJC0112 encoding PNPase-S439-441A with an active site mutant generated by serine to alanine triple mutation at residues 439-441 was generated in pTEV5 using the Gibson assembly method with primers designed by j5 DNA assembly design software. Codons 1-445 of PNPase were amplified by PCR from pMJC0094 with primers MJC222 and MJC261. Codons 437-713 of PNPase were amplified by PCR from pMJC0094 with primers MJC262 and MJC223. Codons 437-445 and the S439-441A mutation were encoded within the 5' ends of primers MJC261 and MJC262 to provide a seamless introduction of the mutation in the PNPase 1-713 ORF in the Gibson reaction. Primers MJC222 and MJC223 include overhang sequences homologous to regions flanking the NheI site in pTEV5. Gibson assembly reaction was completed as previously described resulting in plasmid pMJC0112, which incorporates S439-441A into PNPase. Plasmid pMJC0113 was generated similarly with a modification to the second PCR fragment (PNPase codons 437-713), which was amplified from pMJC0095 with MJC262 and MJC226 to include the C-terminal flexible linker (LRSRAPENSNVTRHRSAT) and mCherry.

Plasmid pMJC0119, RNase CTD PNPase binding site mutant expression vector: pMJC0119 encoding RNase E CTDΔPNPase_BS-YFP contains the C-terminal domain (residues 451-884) of RNase E with a deletion of the PNPase binding site (residues 885-898) and a C-terminal linker (LRSRAPENSNVTRHRSAT) followed by eYFP was generated in pTEV5 using the Gibson assembly method with primers designed by j5 DNA assembly design software. PCR amplified RNase E codons 451-884 from pDT177 with primers MJC265 and MJC280. The linker region and eYFP were amplified by PCR from pDT177 with primers MJC281 and

MJC269. Primers MJC280 and MJC281 include overhang sequences homologous to residues 880-884 and flexible linker codons encoding LRSRAP, and primers MJC265 and MJC269 include overhang sequences homologous to regions flanking the NheI site in pTEV5. As previously described, Gibson assembly was completed, resulting in plasmid pMJC0119, which deletes the PNPase binding site residues from RNase E CTD-YFP.

Plasmid pMJC0137, PNPase-V104A/E224A/F233A-mCherry expression vector: pMJC0137 encoding PNPase-V104A/E224A/F233A-mCherry contains PNPase with alanine mutations to key residues in the RNase E binding pocket of PNPase. pMJC0137 was generated in pTEV5 using the Gibson assembly method with primers designed by j5 DNA assembly design software. Each mutation was successively generated by site-directed mutagenesis using helper plasmids. First, plasmid pMJC0121 was inserted by mutation F233A into PNPase-mCherry using primers MJC282 and MJC283. Second, plasmid pMJC0134 was inserted by mutation V104A into PNPase-F233A-mCherry using primers MJC290 and MJC291. Thirdly, the final plasmid pMJC0137 was made by inserting mutation E224A into PNPase-V104A/F233A-mCherry using primers MJC292 and MJC293. Gibson assembly was completed as described previously.

Plasmid pDT279, MBP-RNase CTD expression vector: pDT279 encoding MBP-RNase E CTD-YFP contains an N-terminal MBP fused to the C-terminal domain (residues 451-898) of RNase E with a C-terminal linker (LRSRAPENSNVTRHRSAT) followed by eYFP was generated in pTEV5 using the TEDA method[5] with primers designed by j5 DNA assembly design software. PCR amplified MBP from pTEV6 (pKLD66)[1] with primers DT471 and DT473. RNase E CTD, a linker region, and eYFP codons were amplified using primers DT476 and DT477. Twenty µL reactions containing 100 mM Tris-Cl pH 7.4, 10 mM $MgCl_2$, 10 mM DTT, 5 wt% PEG(8000), and 0.04 U T5 exonuclease were incubated with a 1:2 vector: insert molar ratio and 100 ng vector.

(1) Rocco, C. J.; Dennison, K. L.; Klenchin, V. A.; Rayment, I.; Escalante-Semerena, J. C. Construction and use of new cloning vectors for the rapid isolation of recombinant proteins from Escherichia coli. *Plasmid* **2008**, *59* (3), 231-237. DOI: 10.1016/j.plasmid.2008.01.001.

(2) Al-Husini, N.; Tomares, D. T.; Bitar, O.; Childers, W. S.; Schrader, J. α-proteobacterial RNA degradosomes assemble liquid-liquid phase-separated RNP bodies. *Mol Cell* **2018**, *71*, 1-13. DOI: 10.1016/j.molcel.2018.08.003.

(3) Gibson, D. G.; Young, L.; Chuang, R. Y.; Venter, J. C.; Hutchison, C. A., 3rd; Smith, H. O. Enzymatic assembly of DNA molecules up to several hundred kilobases. *Nat Methods* **2009**, *6* (5), 343-345. DOI: 10.1038/nmeth.1318.

(4) Chen, J.; Densmore, D.; Ham, T. S.; Keasling, J. D.; Hillson, N. J. DeviceEditor visual biological CAD canvas. *J Biol Eng* **2012**, *6* (1), 1. DOI: 10.1186/1754-1611-6-1. Ham, T. S.; Dmytriv, Z.; Plahar, H.; Chen, J.; Hillson, N. J.; Keasling, J. D. Design, implementation and practice of JBEI-ICE: an open source biological part registry platform and tools. *Nucleic Acids Res* **2012**, *40* (18), e141. DOI: 10.1093/nar/gks531. Hillson, N. J.; Rosengarten, R. D.; Keasling, J. D. j5 DNA assembly design automation software. *ACS Synth Biol* **2012**, *1* (1), 14-21. DOI: 10.1021/sb2000116.

(5) Xia, Y.; Li, K.; Li, J.; Wang, T.; Gu, L.; Xun, L. T5 exonuclease-dependent assembly offers a low-cost method for efficient cloning and site-directed mutagenesis. *Nucleic Acids Res* **2019**, *47* (3), e15. DOI: 10.1093/nar/gky1169.
